# Multidimensional Analysis Integrating Human T-Cell Signatures in Lymphatic Tissues with Sex of Humanized Mice for Prediction of Responses after Dendritic Cell Immunization

**DOI:** 10.3389/fimmu.2017.01709

**Published:** 2017-12-08

**Authors:** Valery Volk, Andreas I. Reppas, Philippe A. Robert, Loukia M. Spineli, Bala Sai Sundarasetty, Sebastian J. Theobald, Andreas Schneider, Laura Gerasch, Candida Deves Roth, Stephan Klöss, Ulrike Koehl, Constantin von Kaisenberg, Constanca Figueiredo, Haralampos Hatzikirou, Michael Meyer-Hermann, Renata Stripecke

**Affiliations:** ^1^Department of Hematology, Hemostasis, Oncology and Stem Cell Transplantation, Hannover Medical School, Hannover, Germany; ^2^Department of Systems Immunology, Braunschweig Integrated Centre of Systems Biology, Helmholtz Centre for Infection Research, Braunschweig, Germany; ^3^Institute of Biostatistics, Hannover Medical School, Hannover, Germany; ^4^Institute of Cellular Therapeutics and GMP Core Facility IFB-Tx, Hannover Medical School, Hannover, Germany; ^5^Clinic of Gynecology and Obstetrics, Hannover Medical School, Hannover, Germany; ^6^Department of Transfusion Medicine, Hannover Medical School, Hannover, Germany

**Keywords:** hematopoietic stem cell transplantation, cord blood, dendritic cell, T cell maturation, lymphatic, humanized mice, gender, artificial neural network

## Abstract

Mice transplanted with human cord blood-derived hematopoietic stem cells (HSCs) became a powerful experimental tool for studying the heterogeneity of human immune reconstitution and immune responses *in vivo*. Yet, analyses of human T cell maturation in humanized models have been hampered by an overall low immune reactivity and lack of methods to define predictive markers of responsiveness. Long-lived human lentiviral induced dendritic cells expressing the cytomegalovirus pp65 protein (iDCpp65) promoted the development of pp65-specific human CD8^+^ T cell responses in NOD.Cg-Rag1^***tm1Mom***^-Il2rγ^***tm1Wj***^ humanized mice through the presentation of immune-dominant antigenic epitopes (signal 1), expression of co-stimulatory molecules (signal 2), and inflammatory cytokines (signal 3). We exploited this validated system to evaluate the effects of mouse sex in the dynamics of T cell homing and maturation status in thymus, blood, bone marrow, spleen, and lymph nodes. Statistical analyses of cell relative frequencies and absolute numbers demonstrated higher CD8^+^ memory T cell reactivity in spleen and lymph nodes of immunized female mice. In order to understand to which extent the multidimensional relation between organ-specific markers predicted the immunization status, the immunophenotypic profiles of individual mice were used to train an artificial neural network designed to discriminate immunized and non-immunized mice. The highest accuracy of immune reactivity prediction could be obtained from lymph node markers of female mice (77.3%). Principal component analyses further identified clusters of markers best suited to describe the heterogeneity of immunization responses *in vivo*. A correlation analysis of these markers reflected a tissue-specific impact of immunization. This allowed for an organ-resolved characterization of the immunization status of individual mice based on the identified set of markers. This new modality of multidimensional analyses can be used as a framework for defining minimal but predictive signatures of human immune responses in mice and suggests critical markers to characterize responses to immunization after HSC transplantation.

## Introduction

Humanized mice transplanted with human hematopoietic stem cells (HSCs) became a broadly used experimental and preclinical platform to characterize the critical steps for the reconstitution of the human immune system (1–3). In this context, humanized mice are currently used to study human-specific infections and to test drugs, vaccines, and cell therapies (2, 3). Engraftment of human HSCs in the mouse bone marrow (BM) and subsequent early T cell development in thymus (Thy) could be conveniently studied in short-term models lasting 10–16 weeks (4). Yet, full maturation of T cells toward memory cells in HSC-transplanted humanized mice was shown to be considerably more heterogeneous and challenging and required periods of analyses of 20 weeks or longer (5, 6). Thus, this lymphopenia coincides with the delayed T cell immune reconstitution in patients after hematopoietic stem cell transplantation (HSCT) (5, 6). Multiple complementary approaches were tried to support the development of human cells in immune-deficient mice such as, for example, the administration of human cytokines (7) and the generation of new transgenic mouse strains expressing human cytokines (8) or human leukocyte antigens (HLA) molecules (9, 10). More complex and demanding strategies exploring co-transplantation with human fetal thymus and liver tissues (bone marrow, liver, thymus model) into mice showed an overall improved T cell development and maturation (11–14). Notably, since T cell responses depend on the strength of the signals delivered by the antigen/HLA to the T cell receptor (TCR) (signal 1), co-stimulation (signal 2), and pro-inflammatory cytokines (signal 3), studies demonstrating the presence of human dendritic cells (DCs) in humanized mice elucidated their role in activation of the cognate T cells (15). Thus, as potential alternative approaches for improving T cell reconstitution in humanized mice and ultimately in humans, adoptive autologous DCs, such as those explored clinically for cancer immunotherapy (16) and human immunodeficiency virus (17), or *in vivo* activated DCs, as previously shown to be effective in humanized mice (18), could represent valuable options. Likewise, we have previously described the preclinical testing of long-lived genetically engineered induced DC (iDCs) in humanized mice. These cells were generated after a fast overnight transduction of monocytes with lentiviral vectors encoding granulocyte-macrophage colony stimulating factor (GM-CSF), interferon-α (IFN-α), and the human cytomegalovirus (HCMV) phosphoprotein (pp) 65 (19, 20). iDCs expressing pp65 (iDCpp65) vaccines are currently in clinical development for protection of posttransplant patients (21), since pp65 has been long known to be a major immune-dominant CD8^+^ cytotoxic T lymphocyte target antigen in healthy seropositive adults (22). Furthermore, non-exhausted, long-lived CD8^+^ effector memory (EM) T cells are considered to be crucial to maintain lifelong protection from HCMV reactivation in posttransplant patients (23).

We previously demonstrated that multiple administrations of iDCpp65 into NOD.Cg-Rag1*^tm1Mom^*-Il2rγ*^tm1Wj^* (NRG) mice transplanted with human HSCs promoted a potent development of CD8^+^ antigen-specific memory responses in short (16 weeks) (20) and long (20–36 weeks) models (19, 24). We have also demonstrated that another important factor to be considered regarding the analyses of human T cells in mice humanized with cord blood (CB)-HSCs is the gender of the recipient mouse. For the initial 10–15 weeks after HSCT, females showed a more robust T cell development and maturation, whereas male’s T cells matched the female’s T cell maturation status only 20 weeks posttransplant (25).

In this current work, we sought to evaluate whether humanized female and male mice would show differential patterns of T cell responses to iDCpp65. We characterized the CD4^+^/CD8^+^ T cells and their subsets [naïve (N), EM, central memory (CM), and terminal effector (TE)] in different lymphatic tissues and confirmed a distinct behavior between females and males, supported by statistical methods. In order to integrate the data obtained from different tissues and evaluate the immunization responsiveness among them, we adopted a classification machine learning algorithm based on an artificial neural network (ANN). A Principal Component Analysis (PCA) (26, 27) was further used to reduce the critical information required to predict responsiveness from the ANN (28). The markers pinpointed by the PCA revealed that the correlation structure of organ-specific markers is strongly impacted by immunization and, therefore, that these markers can be used as biomarkers to retrieve the information of the immunization status.

## Materials and Methods

### Step 1: Generation of Humanized Mice Transplanted with Human CB-HSC

Study protocols were approved by the Ethics Committee of the Hannover Medical School for acquisition and banking of human HSCs obtained from umbilical cord tissues after informed consent from donors (mothers at term). The HSCs were labeled according to a numerical code that could not be traced back to the donor’s personal information, thus keeping the donor’s anonymity. All experiments involving mice were performed in accordance with the regulations and guidelines of the animal welfare of the State of Lower Saxony (Nds. Landesamt für Verbraucherschutz und Lebensmittelsicherheit, Dezernat 33/Tierschutz). 5-week-old NRG mice were originally obtained from The Jackson Laboratory (JAX, Bar Harbor, ME, USA) and bred in-house under pathogen-free conditions. Prior to HSCT, mice were sublethally irradiated (450 cGy) using a [137^Cs^] column irradiator (Gammacell 3000 Elan; Best Theratronics, Ottawa, ON, Canada). 4 h after irradiation, 1.5–2.0 × 10^5^ human CD34^+^ hematopoietic cells isolated from female donor umbilical CB were administrated to each mouse trough the tail vein as described (20, 24). We had previously shown that immune reconstitution in female mice recipients was faster than in males (25) and we, therefore, used female donors to avoid any putative immune responses against antigens expressed in the Y chromosome of male recipients. Stem cells from HLA*A02.01 positive (CB1, CB3) or negative (CB2) units were used to generate humanized mice (CB1: *n* = 11, CB2: *n* = 10, CB3: *n* = 9). Starting at week 10 posttransplantation, the human immune reconstitution in mouse peripheral blood (PB) was assessed by flow cytometry evaluating the frequency of human CD45^+^ cells.

### Step 2: Immunization of Mice with iDCs Expressing the pp65 Antigen

**Figure 1:**
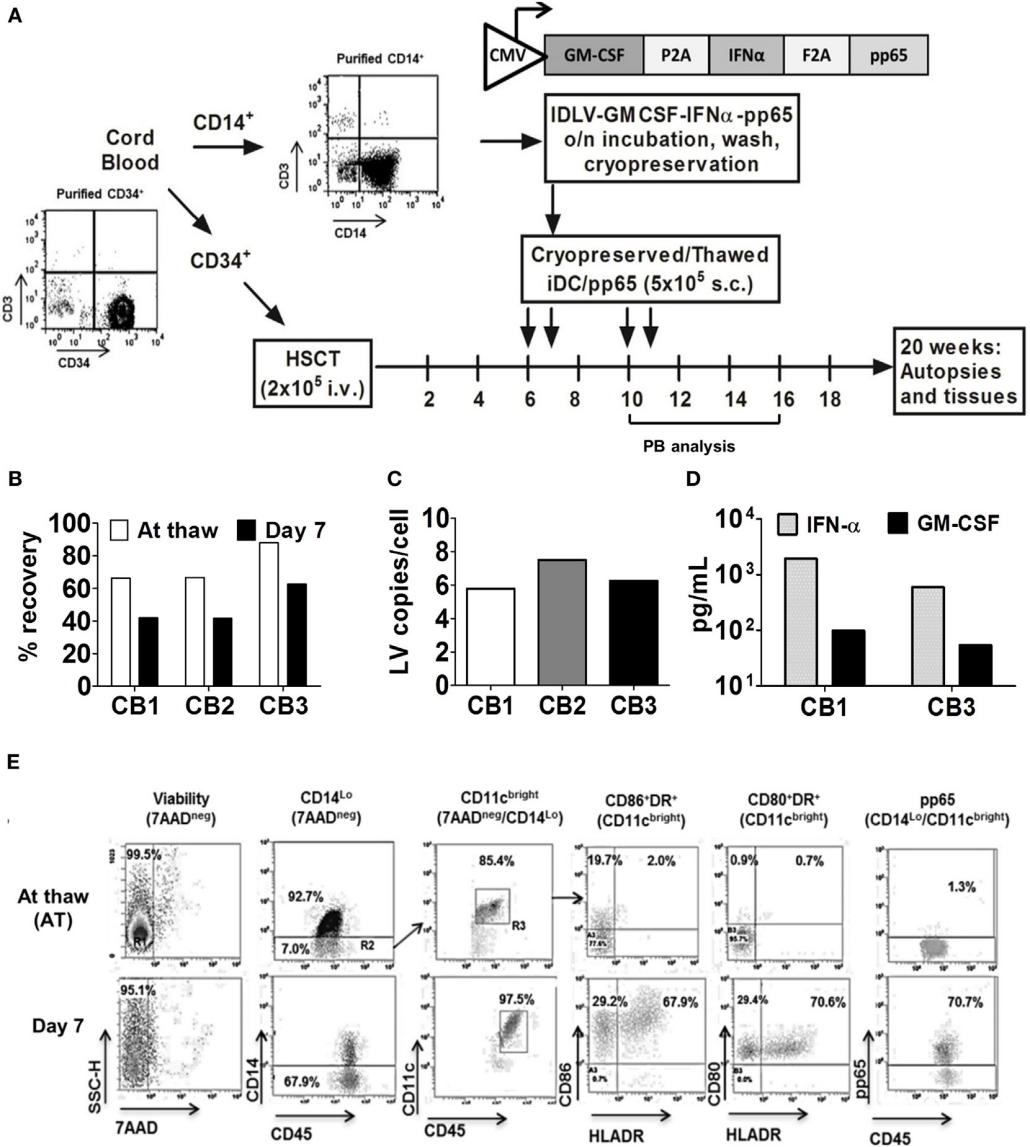
Generation of induced dendritic cells expressing pp65 (iDCpp65) for immunization of humanized mice. **(A)** Scheme of experimental design. Purified CD34^+^ hematopoietic stem cells obtained from three cord blood (CB) units and devoid of contaminating T cells were used for transplantation of three mice cohorts. Purified CD14^+^ cells from the same CB were transduced with a tricistronic integrase-defective lentiviral vector co-expressing huGM-CSF, huIFN-α, and HCMV-pp65. Cryopreserved cells were thawed, analyzed for viability, identity, and potency characteristics *in vitro*, and used for prime/boost immunizations [at weeks 6, 7, 10, and 11 after hematopoietic stem cell transplantation (HSCT), *n* = 17]. Longitudinal analyses of peripheral blood were performed on weeks 10, 16, and 20 after HSCT. Mice were sacrificed at week 20 after HSCT and bone marrow, SPL, Thy, peripheral lymph node (PLN), and MLN were isolated and analyzed. Non-immunized humanized mice (*n* = 11) from the same corresponding CB units were used as controls. **(B)** Percentage of viable iDCpp65 cells after cryopreservation and thawing (white bars) and 7 days after the *in vitro* culture (black bars) for each CB unit. **(C)** iDCpp65 generated with CB 1 (white), 2 (gray), and 3 (black bars) were maintained for 7 days *in vitro* and the extracted DNA was analyzed by RT-q-PCR for LV copy number per cell. **(D)** Concentration of huIFN-α (gray bars) and huGM-CSF (black bars) determined for cell supernatants collected at day 7 of *in vitro* differentiation of iDCpp65 generated from CB donor 1 and 3 and measured by ELISA. **(E)** Representative dot plots of flow cytometry analyses of iDCpp65 (CB1) at thaw and at day 7 of differentiation *in vitro*, showing high viability (7AAD negative population), downregulation of CD14, upregulation of CD45 and CD11c (used as gates for further analyses), and upregulation of HLA-DR, CD80, CD86, and pp65 upon iDCpp65 differentiation.

CD14^+^ monocytes were isolated at high purity (from the same CB units used as source of CD34^+^ HSCs) by immune-magnetic beads (Miltenyi Biotec, Bergisch Gladbach, Germany) and cryopreserved. CD14^+^ cells were used for the generation of iDCpp65 after transduction with a tricistronic integrase-defective lentiviral vector co-expressing human cytokines GM-CSF/IFN-α and the HCMV-pp65 protein as described (IDLV-G2a-pp65) (20, 24). In short, monocytes were pre-conditioned with recombinant human GM-CSF and IL-4 (both 50 ng/ml; Cellgenix, Freiburg, Germany) for 8 h prior to lentiviral gene transfer. Transduction of monocytes with IDLV-G2a-pp65 was performed at a multiplicity of infection of 5 (2.5 mg/ml p24 equivalent) in the presence of 5 µg/ml protamine sulfate (Valeant, Duesseldorf, Germany) for 16 h. Afterward, cells were harvested by resuspension in phosphate-buffered saline (PBS), washed twice, and cryopreserved. For transduction quality assessment, a sample of frozen cells was thawed and maintained in the X-VIVO 15 medium (Lonza, Basel, Switzerland) for 7 days. Analyses of cell viability (by trypan blue exclusion), lentiviral copies per cell (by RT-q-PCR), and DC immunophenotype (by flow cytometry) were performed as previously described (20, 21, 24). For immunization, IDLV-transduced cells were thawed, washed twice with PBS, and re-suspended in PBS at concentration 5.0 × 10^6^ cell/ml. Cells were kept on ice until injection. After CB-HSCT, mice were randomly distributed into two groups, a non-treated control and a group immunized with 5.0 × 10^5^ iDCpp65 cells. Cells were administered subcutaneously in the left hind flank at weeks 6 and 10 and in right hind flank at weeks 7 and 11 posttransplantation (Figure 1A). Weekly weight and general health monitoring were performed until the end of the experiment at week 20 posttransplantation.

### Step 3: Longitudinal Characterization of Human T Cell Development in PB

Samples of PB were collected from all mice at weeks 10, 16, and 20 after HSCT to evaluate the level of engraftment of human hematopoietic cells and expansion of T and B cells. Two rounds of lysis were performed to remove erythrocytes (0.83% ammonium chloride/20 mM HEPES, pH 7.2, for 5 min at room temperature, followed by stabilization with cold PBS and washing). Cells were labeled for flow cytometry analyses with the following antibodies as previously described (24): pacific blue anti-CD45, Alexa700 (AF700) anti-CD19, allophycocyanin (APC) anti-CD3, phycoerythrin-cyanine7 (PC7) anti-CD8 (BioLegend, Fell, Germany); allophycocyanin-H7 (APC-H7) anti-CD4, Phycoerythrin (PE) anti-CD14 (BD Biosciences, San Jose, CA, USA); and phycoerythrin-cyanine5 (PC5) anti-CD62L, fluorescein isothiocyanate (FITC) anti-CD45RA (Beckman Coulter, Krefeld, Germany) for 15 min at room temperature, washed, and analyzed by LSR II flow cytometer (BD Biosciences, Heidelberg, Germany).

### Step 4: Evaluation of the Human T Cell Responses against pp65 in Immunized Mice

Three mice immunized with iDCpp65 and showing well-developed lymph nodes were used for obtaining human memory T cells in high purity as previously described (20, 24). Cryopreserved single cell suspensions generated from lymph nodes were thawed, pooled, and re-suspended in X-VIVO 15 medium. Activation of T cells was performed by MACS magnetic beads conjugated with anti-CD2/CD3/CD28 monoclonal antibodies (Miltenyi Biotec, Germany) in a bead-to-cell ratio of 1:2, in presence of 25 IU/ml of human IL-2, 5 ng/ml IL-7, and 5 ng/ml IL-15 (Cellgenix, Germany) for 48 h. Cytokines were refreshed every 2 days until the end of the homeostatic expansion (day 9 post-activation with magnetic beads). Activated cells were re-stimulated after coculture either with autologous iDC (for APC-mediated homeostatic stimulation but lacking antigens) or with iDCpp65 for 7 days using a T/DC cell ratio of 10:1. For intracellular IFN-γ detection, expanded T cells were first seeded in triplicates wells (3 × 10^5^ cells/well) of a 96-well round-bottom plate and then, for 16 h, the cells were further activated with 10 µg/ml of CMV PepTivator (pp65 overlapping peptide pool, Miltenyi Biotec, Bergisch Gladbach, Germany) or 10 µg/ml of Wilms Tumor 1 (WT1) negative control overlapping peptide pool (Miltenyi Biotec, Germany). A protein transport inhibitor cocktail (eBioscience, Frankfurt, Germany) was added to the cells 2 h after peptide stimulation. At the end of stimulation, the surface staining with APC anti-CD3, APC-H7 anti-CD4, and PC7 anti-CD8 antibodies (BioLegend, Germany) was performed. Subsequently, cells were permeabilized and fixated for intracellular staining with PE anti-IFN-γ antibodies (eBioscience, San Diego, CA, USA). Samples were acquired by LSR II flow cytometer and data were analyzed using FlowJo software version 7.6.4 (Tree Star Inc., Ashland, OR, USA). As targets for enzyme-linked immuno spot (ELISPOT) assays, K562 cells expressing HLA*A02.01 (also known as artificial antigen-presenting cells or “aAPCs”) and aAPCs expressing pp65 endogenously (aAPC/pp65) were cultured in RPMI 1640 (Lonza, Switzerland) containing 10% fetal bovine serum. For IFN-γ detection by ELISPOT, MultiScreen HTS plates (Merk Millipore, Darmstadt, Germany) were coated with anti-IFN-γ antibodies (Mabtech, Nacka Strand, Sweden) at 4°C overnight. Then, 2.5 × 10^4^ T cells were mixed with 7.5 × 10^4^ aAPCs either not carrying an antigen, or with aAPCs pulsed with WT1 peptides (aAPC + WT1), or with aAPCs pulsed with pp65 peptides (aAPC + pp65) or expressing pp65 endogenously (aAPC/pp65). The T cell/aAPC cocultures were incubated overnight, washed, and incubated with biotin-conjugated anti-human IFN-γ monoclonal Ab, followed by incubation with alkaline phosphatase-conjugated streptavidin. Color development was performed using NBT/5-bromo-4-chloro-3-indolyl phosphate liquid substrate, and the plates were analyzed in an ELISPOT reader (AELVIS, Hannover, Germany). The mean ELISPOT counts for activation with iDC (4 replicate cultures) and iDCpp65 (6 replicate cultures) were obtained.

### Step 5: Analyses of Human Cytokines in Mouse Plasma

At sacrifice, PB samples were collected by heart puncture and cells were subsequently sedimented by centrifugation. The supernatant containing plasma was stored at −80°C until the analysis. After thawing, plasma samples were centrifuged at 2,000*g* for 10 min at room temperature prior the analysis to remove remaining cell debris. 25 µl of plasma were used for analysis of each sample. The concentration in plasma of human GM-CSF, IFN-γ, monocyte chemoattractant protein (MCP-1), TNF-α, IL-1β, IL-2, IL-4, IL-5, IL-6, IL-7, IL-8, IL-10, and IL-12 (p70) was analyzed by a 14-plex Luminex kit (Milliplex Millipore, MA, USA) according to manufacturer’s protocol.

### Step 6: Characterization of the Terminal Human T Cell Responses in Lymphatic Tissues

Spleen (SPL), peripheral lymph nodes (PLN), mesenteric lymph nodes (MLNs), BM, and Thy cells were isolated after sacrifice and homogenized. For mice transplanted with CB1-3, cell suspensions generated with PLN and MLN were combined, whereas for CB2 and CB3, there was an additional cell suspension fraction of MLN analyzed separately. Cell suspensions were washed and re-suspended in PBS for subsequent staining with fluorochrome-conjugated monoclonal antibodies as described above for PB (24). Thymocytes were stained 15 min at room temperature with the following antibodies: pacific blue anti-CD45, AF700 anti-CD4, APC anti-CD3, PC7 anti-CD8, FITC anti-TCR αβ (BioLegend, Germany). After washing, cells were re-suspended in PBS containing 1% human serum and analyzed by an LSR II flow cytometer.

### Step 7: Establishing the Database and Statistical Analyses

All variables consisting of cell phenotypes determined as relative frequency (PB and lymphatic tissues) and counts (lymphatic tissues) and cytokines concentrations (plasma) were organized in a PivotTable using Excel software 2010 (Microsoft, Redmond, WA, USA). Each sample was coded according to the CB unit (1, 2, or 3) cell type, tissue, time-point of analyses, intervention group (control or iDCpp65), and mouse gender (female “F” or male “M”). Beta regression analysis was employed to model the association between the intervention groups and the cell phenotypes measured as “relative frequency,” which is expressed as odds ratio (i.e., the odds of event between iDCpp65 and control), whereas negative binomial regression was used to model the association between the intervention groups and the count cell phenotypes expressed as rate ratio (i.e., the incidence rate between iDCpp65 and control). Both models where implemented with and without stratification by gender. Parameter estimation was performed by least square means. The *p*-values calculated at significance levels 0.05 and 0.01 using the two-sided *z*-test statistic were considered significant. All analyses were implemented using the SAS 9.3 software (SAS, Cary, NC, USA). PROC GLIMMIX was used for Beta regression analysis, together with PROC GENMOD for the negative binomial regression analysis. A two-way analysis of variance was performed to analyze the results of the IFN-γ ELISPOT assay using GraphPad Prism version 5 software (GraphPad Software, Inc., La Jolla, CA, USA).

### Step 8: ANN Classification Approach

The computational analysis was carried out in MatLab version 7.11.0, 2010 (MathWorks, Inc., Natick, MA, USA) using the Neural Network Pattern recognition application. The inputs (15 markers representing cell phenotypes for PB, SPL, PLN + MLN, or MLN, BM and 8 markers for Thy) and their corresponding outputs [immunization status of each mice, “yes” (1) or “no” (0)] were used for training the ANN. To find the appropriate number of neurons in the hidden layer, we performed a *k*-fold cross validation (29) (with *k* being equal to 3, 4, 5) resulting in 12 neurons (8 for Thy). The output layer consisted of two neurons. The classification accuracy (percentage of correct classifications) was estimated by averaging over 2000 ANN. For each of those ANN, the input dataset was randomly divided into training (70% samples/tissue), validation (15% samples) and testing (15% samples). The Levenberg–Marquardt back-propagation (trainlm) algorithm was used for the training step, as it is best suited for small networks (30). For the hidden layer, we used sigmoid transfer functions. The data were first standardized (i.e., each marker was normalized to 0 mean value and unit variance) in order all the markers to be at the same scale.

We also performed the same classification analysis of immunized (1) and non-immunized mice (0), but by dividing our samples into male and female mice data sets. In this case, we used the same parameter setting for ANN as above and tested the classification accuracy for each subpopulation. In addition to the classification accuracy (defined as the fraction of correctly classified samples), we measured the sensitivity and specificity of the classification, applied inside each group. The *sensitivity*, defined as

$$\text{sensitivity}=\frac{\text{number of true positives}}{\text{number of true positives}+\text{number of false negatives}},$$

represents the probability of a sample classified as immunized (1) to belong to the immunized group.

The *specificity* provides the probability of a sample classified as non-immunized (0) to belong to the non-immunized group. Specificity is defined as

$$\text{specificity}=\frac{\text{number of true negatives}}{\text{number of true negatives}+\text{number of false positives}}.$$

The 15 markers correspond to the frequencies of human lymphocyte lineages analyzed per tissue (PB, BM, SPL, MLN + PLN, MLN) of each mouse included cells determined as frequencies of CD45, CD19/CD45, CD3/CD45, CD14/CD45 (in BM CD34/CD45 instead of CD14/CD45), CD4/CD45, CD8/CD45 and a frequency of other non-determined CD45^+^ cells, frequencies of CD4 subtypes (N, CM, EM, and TE), and frequencies of CD8 subtypes (N, CM, EM, and TE). Eight markers were used for Thy: frequencies of CD45, CD3/CD45, CD4/CD45, CD8/CD45, CD4^−^/CD8^−^ (double negative, DN), CD4^+^/CD8^+^ (double positive, DP), TCRαβ^+^/CD3, and CD4 to CD8 ratio values.

### Step 9: PCA

Principal component analysis (31) was used to recognize clusters of interrelated markers for control and iDCpp65 mice in the different tissues. PCA constructs specific directions, which are called principal components, along which the data are most dispersed and thus best distinguishable. In this way, a data set can be represented by the principal components, which incorporate a specific amount of the variance (or dispersion). Since the components are uncorrelated of each other, the markers, which are strongly correlated with a component, compose a cluster of markers, which vary together. Here, we created these clusters by selecting the phenotypic markers that were strongly correlated or anti-correlated (more than 80%) with any component for each group. The first four components were considered for analysis since they were able to incorporate more than 80% of the total variance in both control and iDCpp65 mice in all tissues. These components were the basis of variance-distribution comparisons and correlation heat-maps for both mouse groups. The markers that were strongly correlated or anti-correlated with the first governing component were used for correlation comparisons between the control and iDCpp65 mice. The data for control and iDCpp65 mice from BM and SPL were represented by 30 variables (percentages and counts for each of the 15 markers); from combined PLN, PB, and MLN by 15 variables (only percentages); from Thy by 14 variables (percentages and counts). As in the training of ANN, the data were first standardized (i.e., each marker was normalized to 0 mean value and unit variance) in order renormalize all markers to the same scale.

## Results

### Cryopreserved iDCpp65 Remained Viability and Characteristics after Thawing

We have shown before that human T cell responses against HCMV-pp65 were consistently stimulated in humanized NRG mice immunized with iDCpp65 (19, 20, 24). Here, iDCpp65 were generated and cryopreserved immediately after transduction, and subsequently used for prime-boost immunizations of female and male mice (Figure 1A). Effects of immunizations in lymphatic tissues were analyzed 20 weeks after HSCT corresponding to 9 weeks after the last immunization. The cell vaccine, iDCpp65, showed high viability directly after thawing (66–88%) and *in vitro* culture for 7 days (42–63%) relative to starting number of cells (Figure 1B). Efficient transduction with IDLV and persistency of episomal viral copies were confirmed for cells maintained in culture for 7 days and showing in average five lentiviral copies/cell (Figure 1C). Transgenic cytokines that accumulated on the cell supernatant of iDCpp65 derived from CB1 and CB3 for 7 days were detected in the range of 500–1,000 pg/ml for IFN-α and 50–100 pg/ml for GM-CSF (Figure 1D). The viable cells showed a typical DC immunophenotype with co-expression of HLA-DR (61.60–91.85%), CD86 (93.20–98.97%), and CD80 (29.1–97.7%) surface markers (Figure 1E, representative data of a batch of iDCpp65, Figure S1A in Supplementary Material). Intracellular immunostaining for detection of pp65-positive iDCpp65 cells by flow cytometry assay showed variable results among different CB donors (CB1: 49.20%, CB2: 2.49%, CB3: 16.40% when calculated for total viable cells in suspension 7 days after *in vitro* culture, Figure S1B in Supplementary Material).

### iDCpp65 Immunizations Affected Lymphocytes Counts in PB, Plasma Cytokines Profiles, and Lymph Node Development

**Figure 2:**
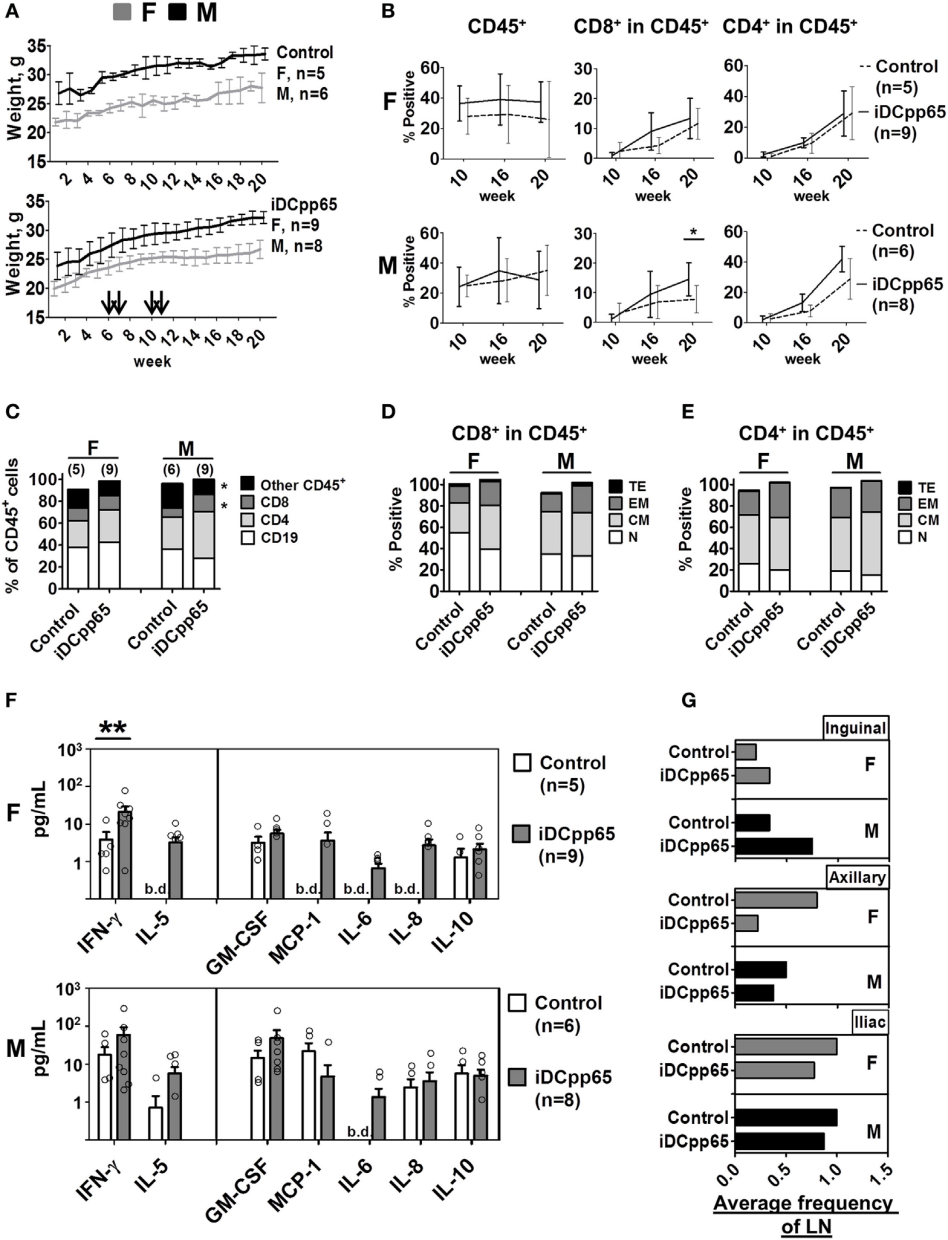
Longitudinal analyses of female and male humanized mice. **(A)** Weight monitoring of control (*n* = 11, upper panel) and immunized mice (*n* = 17, lower panel). Arrows indicate the weeks of induced dendritic cells expressing pp65 (iDCpp65) immunizations after hematopoietic stem cell transplantation. Bi-weekly weight (g) determined for females (F) indicated in gray and for males (M) in black. The number of F and M mice per group is indicated. **(B)** Mean relative frequencies of human CD45^+^, CD8^+^ in CD45^+^, and CD4^+^ in CD45^+^ cells determined in blood by flow cytometry of control (dashed line) and iDCpp65-immunized (solid line) F (upper panel) and M (lower panel) at weeks 10, 16, and 20 posttransplantation. Error bars represent SEs. *p < 0.05 are indicated on the graph. **(C)** Relative frequencies of human cell types measured by flow cytometry in PBL of female (F) and male (M) mice at week 20 posttransplantation. Mean relative frequencies of CD19^+^ (white), CD4^+^ (light gray), CD8^+^ (dark gray), and other CD45^+^ cells (black) were measured in control and iDCpp65-immunized mice, *p < 0.05 are indicated on the graph. **(D)** Phenotypes of distinct CD8^+^ and **(E)** CD4^+^ T cells were determined as naïve (N, white, CD45RA^+^/CD62L^+^), central memory (CM, light gray, CD45RA^−^/CD62L^+^), effector memory (EM, dark gray, CD45RA^−^/CD62L^−^) and terminal effector (TE, black, CD45RA^+^/CD62L^−^). Mean relative frequencies are shown for control and iDCpp65-immunized mice. **(F)** Concentration of human cytokines measured in plasma of F (upper panel) and M (lower panel) mice 20 weeks posttransplantation. Mean concentrations of cytokines were determined for iDCpp65-immunized (gray) and control (white) groups. Bars and circles reflect the SE of the estimated mean concentrations and the observed concentrations of individual samples, respectively. Concentration values below the detection limit of the assay are indicated (b.d.), *p < 0.05, **p < 0.01 indicated on the graph. **(G)** The average frequency of lymph nodes found in F (top panel) and in M (bottom panel) mice for iDCpp65-immunized and control groups. Values for inguinal (upper-), axillary (middle-), and iliac (lower panel) lymph nodes found 20 weeks after hematopoietic stem cell transplantation. Number of samples: female, *n* = 5/9; male, *n* = 6/8, control/immunized, respectively.

Three independent cohorts of NRG mice after CD34^+^ HSCT were generated. 5.0 × 10^5^ thawed and viable autologous iDCpp65 were injected at weeks 6, 7, 10, and 11 after HSCT. Body-weight and general health conditions were monitored weekly. Although females from both control and iDCpp65-immunized cohorts were lighter than males, mice of both genders gained weight normally for the 20 weeks after HSCT (Figure 2A) and showed no signs of graft-versus-host disease or organ pathologies at sacrifice (data not shown). The frequencies of human CD45^+^ cells detected in PB at weeks 10, 16, and 20 post-HSCT were consistently higher in females, particularly for females immunized with iDCpp65 (Figure 2B). The expansion of human CD8^+^ and CD4^+^ T cell detectable in PB was superior in the iDCpp65 cohort compared to controls, especially in the male group (Figure 2B; Table S1 in Supplementary Material). Analyses of human cells in PB 20 weeks after HSCT by assessment of the mean relative frequencies showed that CD8^+^ T cells were higher in the immunized cohort (*p* = 0.03) (Table S1 in Supplementary Material), with high significance for the male group (*p* = 0.01 CD8^+^; *p* = 0.09 CD4^+^) (Figure 2C; Table S1 in Supplementary Material). This was associated with a higher accumulation of EM and TE CD8^+^ cells in the group of iDCpp65-immunized males whereas the group of females showed higher accumulation of CM CD8^+^ cells in immunized versus control group (Figure 2D; Table S1 in Supplementary Material). The relative frequencies of CD4^+^ T cell subtypes were only slightly altered, showing an increase in the relative frequencies of CM cells for males and EM cells for females (Figure 2E; Table S1 in Supplementary Material). A fluorescent-based bead assay was used to measure the concentration of 12 human cytokines in mouse plasma (GM-CSF, MCP-1, IFN-γ, TNF-α, IL-1β, IL-2, IL-4, IL-5, IL-6, IL-8, IL-10, and IL-12p70). IL-1β, IL-2, IL-4, and IL-12p70 were below the detection limit. For the group of non-immunized mice, the baseline concentrations of human cytokines in the plasma were consistently higher for males. Remarkably, IL-5, MCP-1, and IL-8 were only detectable in non-immunized males. Upon immunizations, both genders showed increased IFN-γ (rate ratio 2.74, *p* = 0.15) and GM-CSF concentrations (rate ratio 2.96, *p* = 0.092) (Table S2 in Supplementary Material). The increase of IFN-γ concentration after immunization was particularly high for immunized females (*p* = 0.003) (Figure 2F). Detection of IL-5, MCP-1, IL-6, and IL-8, in plasma of females was only possible after iDCpp65 immunization. Therefore, overall, iDCpp65 immunization harnessed the maturation of human T cells in PB, which was in general associated with an increase of human cytokines in plasma (in particular for females). The undersized and incompletely developed lymph nodes in humanized mice are difficult to be detected. They can be, nonetheless, detected as quite small “fatty” structures in the expected anatomical regions (inguinal, axillary, iliac). Upon immunization with iDCpp65, these draining LNs become macroscopically more noticeable. Although these regenerated LNs are not fully “normal” in relationship to lymph nodes found in immune competent mice, they contain a high density of human T cells (19). In this current study, we confirmed a higher frequency of developed draining lymph nodes (near the immunization sites) in the immunized cohorts, which was more evident for males (Figure 2G). Remarkably, this observation was inverted for the axillary lymph nodes, which were more prominent in females. On the other hand, the average frequency of detectable iliac nodes was lower in the immunized cohort (Figure 2G). Small MLNs developed in most mice, regardless of gender or immunizations (data not shown), indicating the possibility that the development of MLNs may be induced differently.

### iDCpp65-Immunized Mice Demonstrated Functional pp65-Specific Memory T Cell Responses

**Figure 3:**
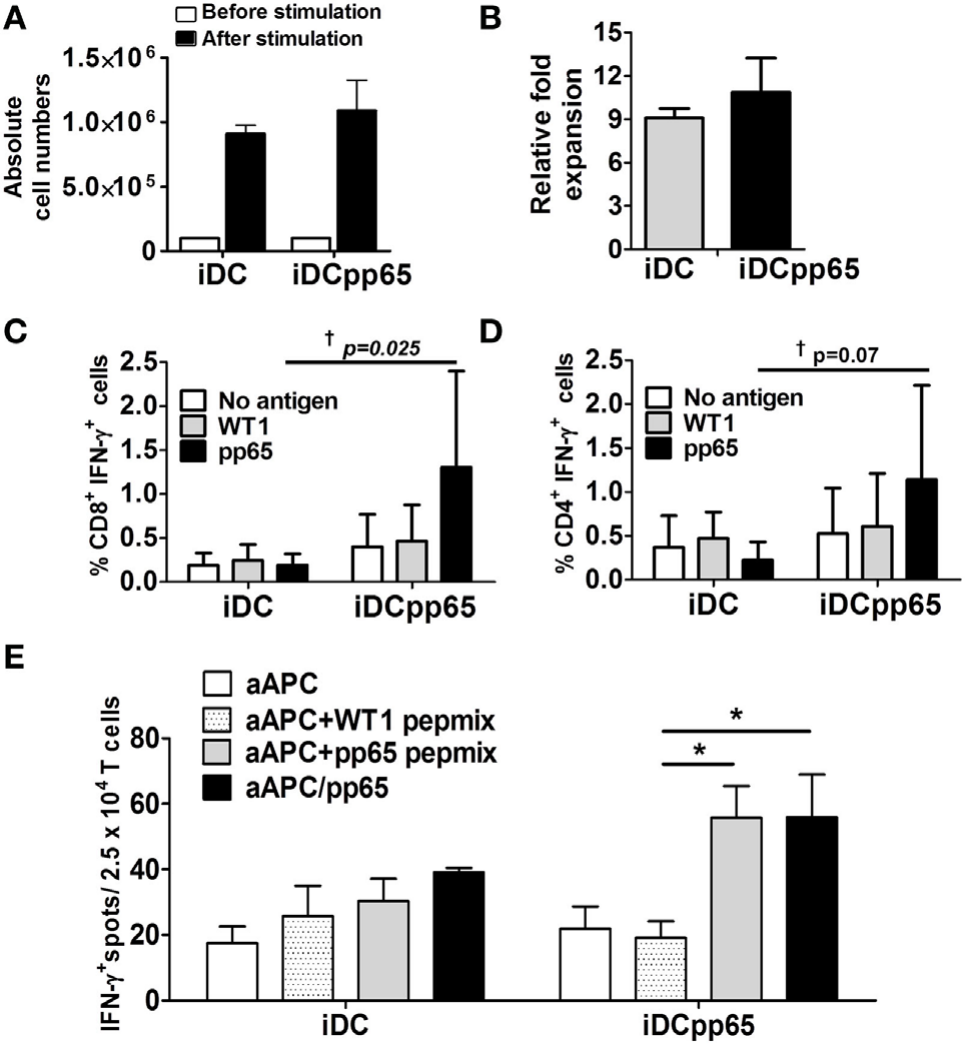
Functional memory T responses against pp65 after induced dendritic cells expressing pp65 (iDCpp65) immunization. **(A)** T cells isolated from lymph nodes of immunized mice (*n* = 3) were re-stimulated *in vitro* with either induced DC (iDC) or iDCpp65. Absolute T cell numbers before (white bars) and 7 days after (black bars) stimulation are shown. **(B)** Relative fold increase of cells populations before and 7 days after coculture with iDC (gray bars) and iDCpp65 group (black bar). **(C)** CD8^+^ and **(D)** CD4^+^ T lymphocytes expanded after coculture with iDC (*n* = 3) or iDCpp65 (*n* = 3) were left unstimulated (white bars) or re-stimulated with a Wilms Tumor 1 (WT1) peptide pool (gray bars) or pp65-peptide pool (black bars). Frequency of cells producing IFN-γ is shown. **(E)** T cells expanded with iDC (four cultures per group) or iDCpp65 (six cultures per group) were cocultured with an artificial antigen-presenting cell (aAPC) on an ELISPOT plate. The aAPCs were ether not loaded (white bars), loaded with WT1 peptides (light gray bars), loaded with pp65-peptides (dark gray bars), or transduced for endogenous pp65 expression and loading (black bars). Mean absolute number of IFN-γ-spots and *p < 0.05 (analysis of variance), ^†^*p* < 0.05 (*F*-test) are indicated.

Lymph nodes of immunized mice were a valuable compartment for the detection of high frequencies of human T cells. Lymphocytes recovered from lymph nodes of three immunized mice were pooled and maintained *in vitro* for 48 h for homeostatic activation by beads and cytokines. Lymphocytes from non-immunized mice were not used since, from previous experience, we were not able to expand them successfully *in vitro* (19, 20). Microcultures of cell suspensions were incubated with autologous “empty” iDCs or with iDCpp65 at 10:1 T to DC ratio for 1 week to promote further expansion of T cells. The expansion was more pronounced for T cells cultured in the presence of iDCpp65 stimulation than “empty” iDCs (fold expansion relative the population before stimulation): 10.9 (iDCpp65) and 9.1 (iDCs) (Figures 3A,B). Activated CD8^+^ and CD4^+^ T cells were analyzed by flow cytometry for detection of intracellular IFN-γ. T cells expanded in the presence of empty iDC and re-stimulated with WT1 or pp65-peptide pools showed similar baseline frequencies of CD8^+^ IFN-γ^+^ and CD4^+^ IFN-γ^+^ T cells. In contrast, T cells expanded in the presence of iDCpp65 and then re-stimulated with the pp65-peptide pool showed variable but in average much higher relative frequencies of CD8^+^ IFN-γ^+^ and CD4^+^ IFN-γ^+^ T cells (Figures 3C,D). The mean anti-pp65 response for CD8^+^ was 6.84 times higher in the iDCpp65 than in iDC re-stimulation group and for CD4^+^ T cells in 6.27 times, respectively. F-test comparing variances between iDC and iDCpp65 showed a strong evidence of variance difference between groups in case of re-stimulation with pp65-peptide pool (*p* = 0.025, CD8^+^; *p* = 0.072, CD4^+^) but not when groups were re-stimulated with WT1 or not stimulated at all (*p* > 0.05 for all cases) (Figures 3C,D).

As a complementary approach, the ability of T cells to recognize and be activated by pp65 epitopes presented by an aAPC positive for HLA-A*02.01 was tested by an IFN-γ-ELISPOT assay as previously described (32). T cells expanded after coculture with iDCs or iDCpp65 were exposed overnight to different types of aAPCs, and the numbers of reactive T cells were quantified. T cells expanded with iDCpp65 and cocultured with either aAPC loaded with pp65-peptides or transduced for pp65 expression showed on average significantly higher frequencies of activated T cells than when cocultured with aAPC loaded with control WT1 peptides (*p* = 0.016, *p* = 0.026, respectively). No significant amplification of T cell activation was observed when T cells were expanded in the presence of “empty” iDCs (*p* > 0.05 for both cases) (Figure 3E). These data confirmed that immunizations with iDCpp65 promoted a specific immune competence against pp65 in humanized mice which was mediated by human T cells.

### Heterogeneous Patterns of Human Lymphocytes in Lymphatic Tissues after iDCpp65 Immunization

**Figure 4:**
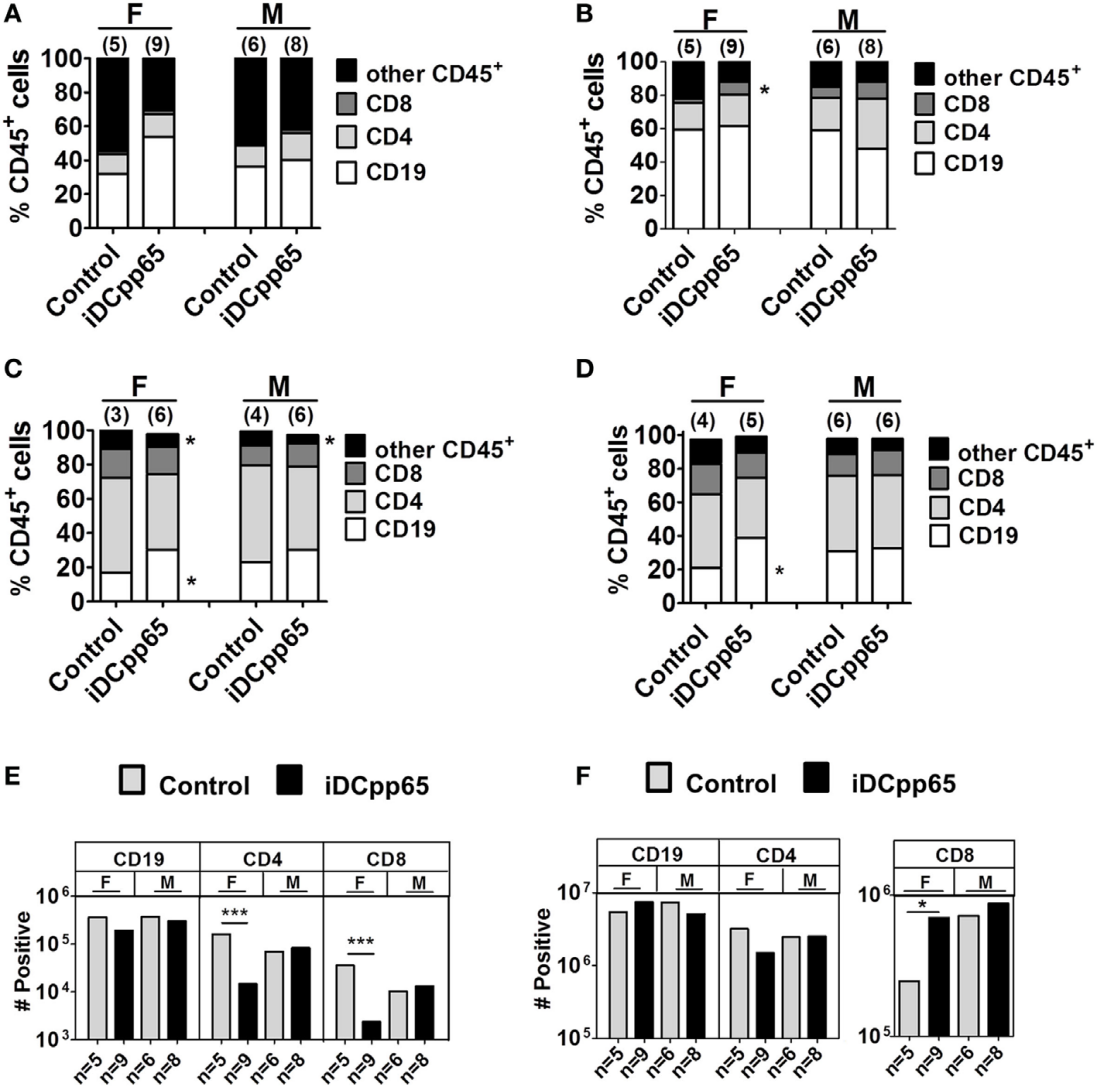
Relative and absolute quantification of human hematopoietic lineages in lymphatic tissues. Mean relative frequency for human CD19^+^ (white), CD4^+^ (light gray), CD8^+^ (dark gray), and other CD45^+^ cells (black) detected by flow cytometry analyses of **(A)** bone marrow, **(B)** SPL, **(C)** combined peripheral lymph nodes and MLN, and **(D)** MLN. Frequencies for female (F) and male (M) mice are shown separately. **(E)** Absolute cell counts for bone marrow and **(F)** spleen were obtained. Plots represent average number of cells determined for control (gray bars) and induced dendritic cells expressing pp65 (iDCpp65)-immunized (black bars) split between female and male mice. The number of mice analyzed per group (*n*) and *p < 0.05 is indicated on the graph. For **(A,B,E,F)** F/control *n* = 5, F/iDCpp65 *n* = 9, M/control *n* = 6, M/iDCpp65 *n* = 8. For **(C)** F/control *n* = 3, F/iDCpp65 *n* = 6, M/control *n* = 4, M/iDCpp65 *n* = 6. For **(D)** F/control *n* = 4, F/iDCpp65 *n* = 5, M/control *n* = 6, M/iDCpp65 *n* = 6.

Isolated tissues [BM, SPL, lymph nodes (PLN + MLN and MLN)] were processed for flow cytometry analyses and quantification of human lymphocyte frequencies and absolute numbers. For BM, CD3^+^ T cells represented only a minority of huCD45^+^ cells, whereas B cells (CD19^+^) and other CD45^+^ cells prevailed. In terms of relative frequencies, a trend for increased frequencies of CD19^+^ cells was observed upon iDCpp65 immunization, an effect that was more pronounced in females (Figure 4A; Table S3 in Supplementary Material). Remarkably, the total number of BM cells was consistently lower in the immunized group, which reflected into a noticeable lower absolute number of T cells, especially for the female group (Figure 4E; Table S3 in Supplementary Material). This was offset by analysis of splenocytes, showing an overall higher relative frequency and absolute counts of CD8^+^ cells within the huCD45^+^ population of immunized compared with control mice, especially for females (Figures 4B,F; Table S4 in Supplementary Material). As a consequence of the variable detection of lymph nodes in host mice, the analyses of absolute cell numbers also varied accordingly. Lymph nodes from different body parts were initially combined for CB1, but as the development and functions of PLNs (axillary, brachial, inguinal, and iliac) seemed to be distinct from that of the MLN, they were further separately analyzed into two independent groups for CB2 and CB3. Nevertheless, with either combining the lymph nodes or analyzing MLN separately, the general trend was increased frequencies of CD19^+^ cells upon immunization and was more pronounced in females (Figures 4C,D). Concurrently, a relative decrease in the frequency of T cells was observed (Figures 4C,D; Table S5 in Supplementary Material). This data indicated that the patterns of different lymphocyte types were heterogeneous and largely influenced by the tissue analyzed, sex of the hosts, and whether they were immunized or not.

### The Patterns of T Cell Maturation after iDCpp65 Immunization Varied in Lymphatic Tissues

**Figure 5:**
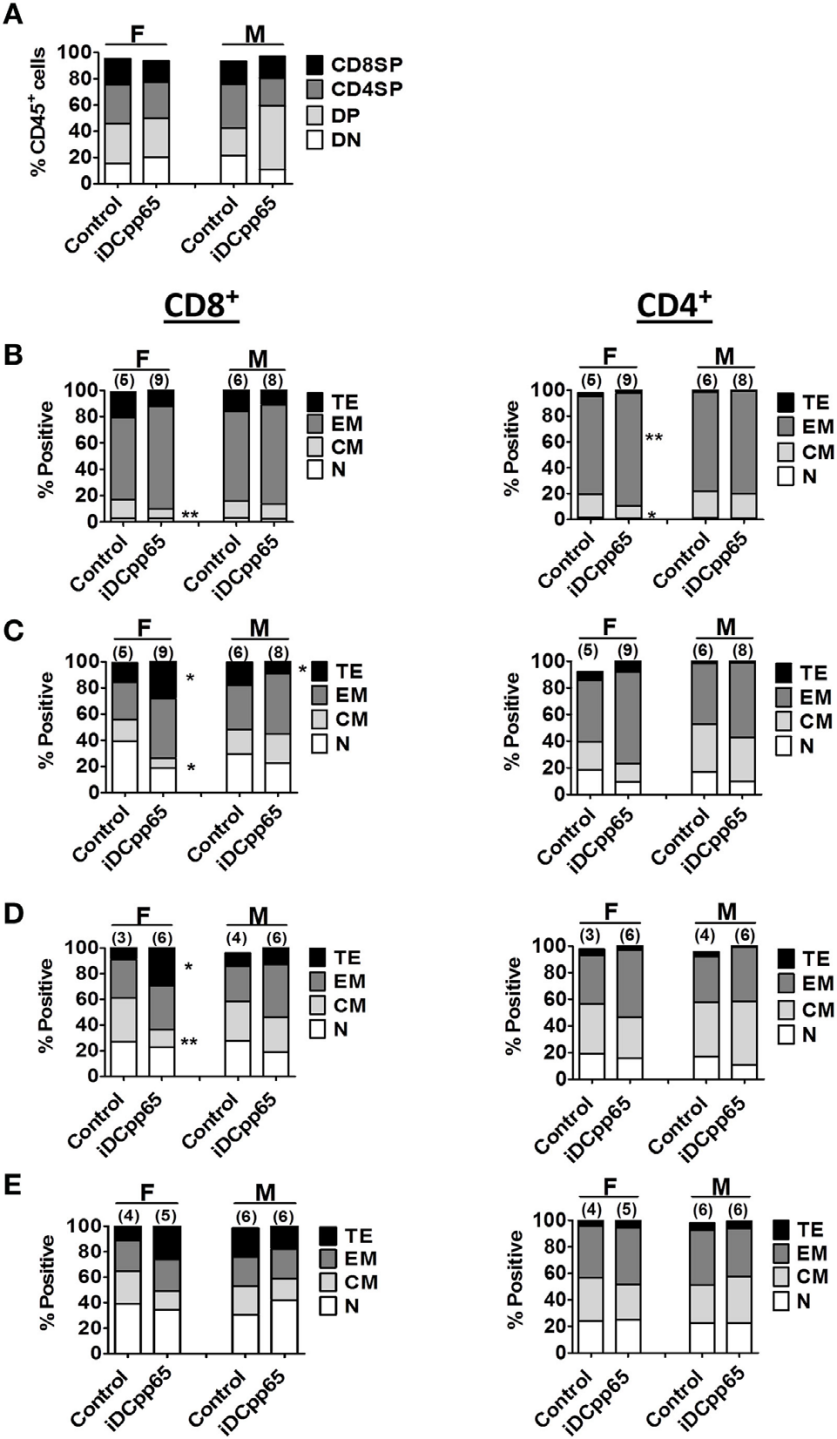
Relative quantification of human T cell subtypes in lymphatic tissues. Mean relative frequencies of human T cell subtypes were estimated based on results of flow cytometry analyses 20 weeks posttransplantation for F and M mice. **(A)** Mean relative frequencies of thymic CD4/CD8 double negative (DN, white), CD4/CD8 double-positive (DP, light gray), CD4^+^ single-positive (CD4SP, dark gray), and CD8^+^ single-positive (CD8SP, black) cells within huCD45^+^ cells in control and induced dendritic cells expressing pp65 (iDCpp65)-immunized mice. Phenotypes of distinct CD8^+^ and CD4^+^ T cells subtypes: **(B)** bone marrow, **(C)** SPL, **(D)** combined PLN and MLN, and **(E)** MLN. Subtypes were determined as Naïve (N, white, CD45RA^+^/CD62L^+^), central memory (CM, light gray, CD45RA^−^/CD62L^+^), effector memory (EM, dark gray, CD45RA^−^/CD62L^−^), and terminal effector (TE, black, CD45RA^+^/CD62L^−^). Mean relative frequencies are shown for control and iDCpp65-immunized mice. The sample size for female and male mice and *p < 0.05, **p < 0.01 are indicated on the graph. For **(A)** F/control *n* = 5, F/iDCpp65 *n* = 9, M/control *n* = 6, M/iDCpp65 *n* = 7. For **(B,C)** F/control *n* = 6, F/iDCpp65 *n* = 9, M/control *n* = 6, M/iDCpp65 *n* = 8. For **(D)** F/control *n* = 3, F/iDCpp65 *n* = 6, M/control *n* = 4, M/iDCpp65 *n* = 6. For **(E)** F/control *n* = 4, F/iDCpp65 *n* = 5, M/control *n* = 6, M/iDCpp65 *n* = 6.

**Figure 6:**
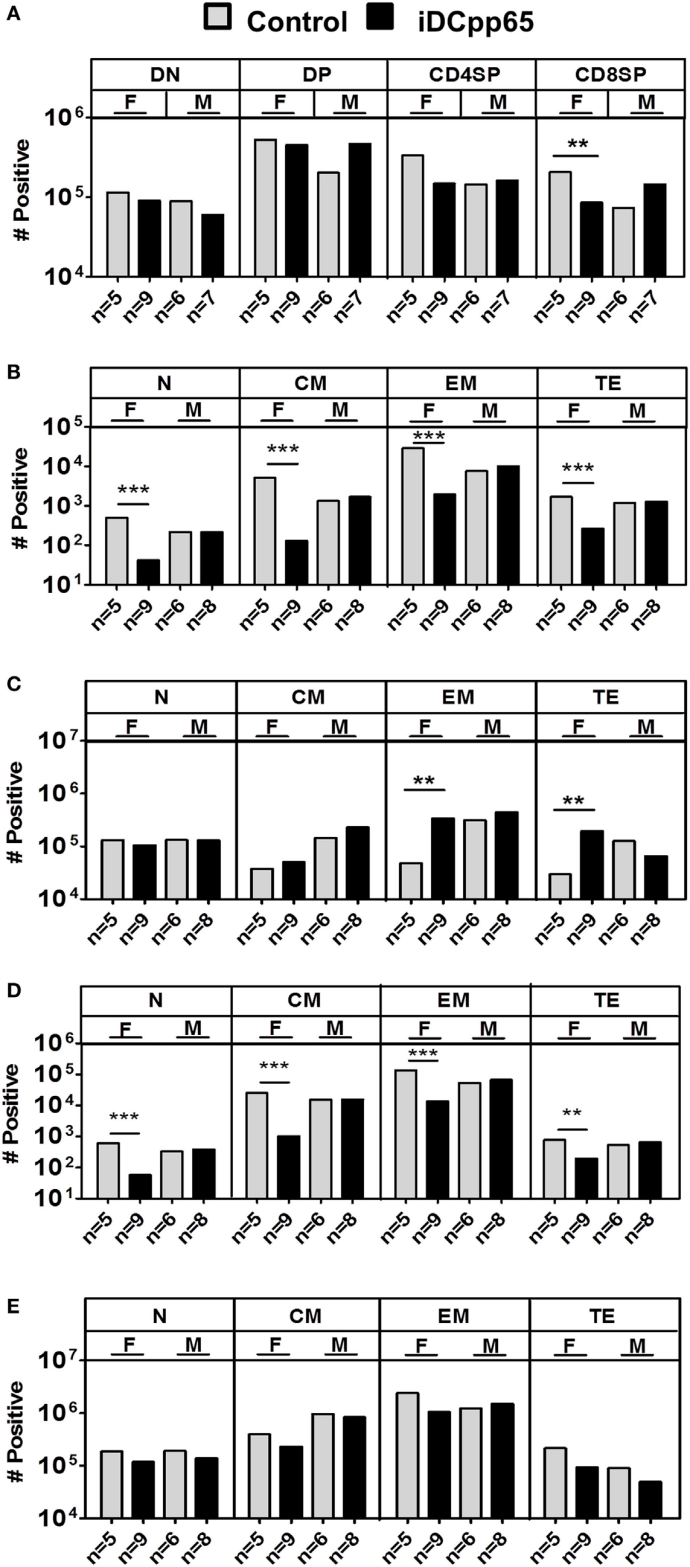
Absolute cell counts of T cell subtypes in different tissues. Mean cell counts determined for female (F) and male (M) mice 20 weeks posttransplantation. **(A)** Analyses of Thy showing control (gray bars) and induced dendritic cells expressing pp65 (iDCpp65)-immunized (black bars) groups. **(B)** Mean cell counts of CD8^+^ T cell subtypes determined in bone marrow (BM) and **(C)** SPL of F and M mice for control and iDCpp65-immunized groups. **(D)** Mean cell counts of CD4^+^ T cell subtypes determined in BM and **(E)** SPL of F and M mice for control and iDCpp65-immunized groups. Subtypes were determined as Naïve (N, CD45RA^+^/CD62L^+^), central memory (CM, CD45RA^−^/CD62L^+^), effector memory (EM, CD45RA^−^/CD62L^−^), and terminal effector (TE, CD45RA^+^/CD62L^−^). Mean relative frequencies are shown for control and iDCpp65-immunized mice. The sample size for females and males and **p < 0.01 are indicated on the graph. **(A)** F/control *n* = 5, F/iDCpp65 *n* = 9, M/control *n* = 6, M/iDCpp65 *n* = 7. For **(B–E)** F/control *n* = 5, F/iDCpp65 *n* = 9, M/control *n* = 6, M/iDCpp65 *n* = 8.

Analysis of CD4^+^, CD8^+^, double positive (DP) and double negative (DN) T cells in Thy showed just a modest higher relative frequency and absolute counts of DP cells for iDCpp65-immunized mice, but only in male group (Figure 5A; Table S6 in Supplementary Material). Notably, females showed reduced absolute counts of single-positive CD8^+^ cells in the immunized group (Figure 6A; Table S6 in Supplementary Material). For the BM, the most abundant T cell subtypes were EM (Figures 5B and 6B,D). The CD8^+^ and CD4^+^ EM T cell frequencies were further augmented upon immunization, but only for the female group, which also resulted in the relative decrease of CM CD8^+^ and CD4^+^ cells (Figure 5B; Table S3 in Supplementary Material). For T cells in SPL, immunization with iDCpp65 resulted in a greater proportion of CD8EM and CD4EM T cell subtypes compared to control mice (Table S4 in Supplementary Material). Females demonstrated a more abundant accumulation of mature EM and TE CD8^+^ cells (Figures 5C and 6C,E; Table S4 in Supplementary Material). The analysis of T cell phenotypes in combined PLN and MLN revealed a skewing toward EM among CD8^+^ and CD4^+^ cells (Table S5 in Supplementary Material), notably in females for the CD8TE subtype (*p* = 0.047) upon immunization (Figure 5D; Table S5 in Supplementary Material). A separate analysis was performed for MLN only and similarly showed the trend toward accumulation of mature CD8TE cells upon immunization (*p* = 0.06) specifically for the female group (Figure 5E; Table S5 in Supplementary Material). These data confirmed that immunizations with iDCpp65 affected T cells and promoted their conversion toward more mature subtypes.

### A Machine Learning-Based Predictive Classifier of Immunized and Non-Immunized Mice

In the previous parts, the comparison of single markers to find phenotypic immunological parameters affected by immunization with iDCpp65 showed that the profile and magnitude of these immunization-influenced parameters were very heterogeneous among the analyzed tissues (Figures 2 and 4–6). In order to provide an integrative view on how immunizations impacted different tissues, including how the immune-phenotypic markers were correlated between the control and the immunized group, a multidimensional analysis was established.

In a first approach, and in order to understand which parts of the multidimensional immune response in different tissues contained the critical information about the responsiveness to immunization, we asked whether a classification between control and iDCpp65 samples could be achieved in each tissue by employing an ANN. We investigated whether the cellular composition of single organs would characterize the response to iDCpp65 immunization. To this end, we measured the potency of ANN to recognize any patterns associated with iDCpp65 immunization and screened for them among different tissues.

**Figure 7:**
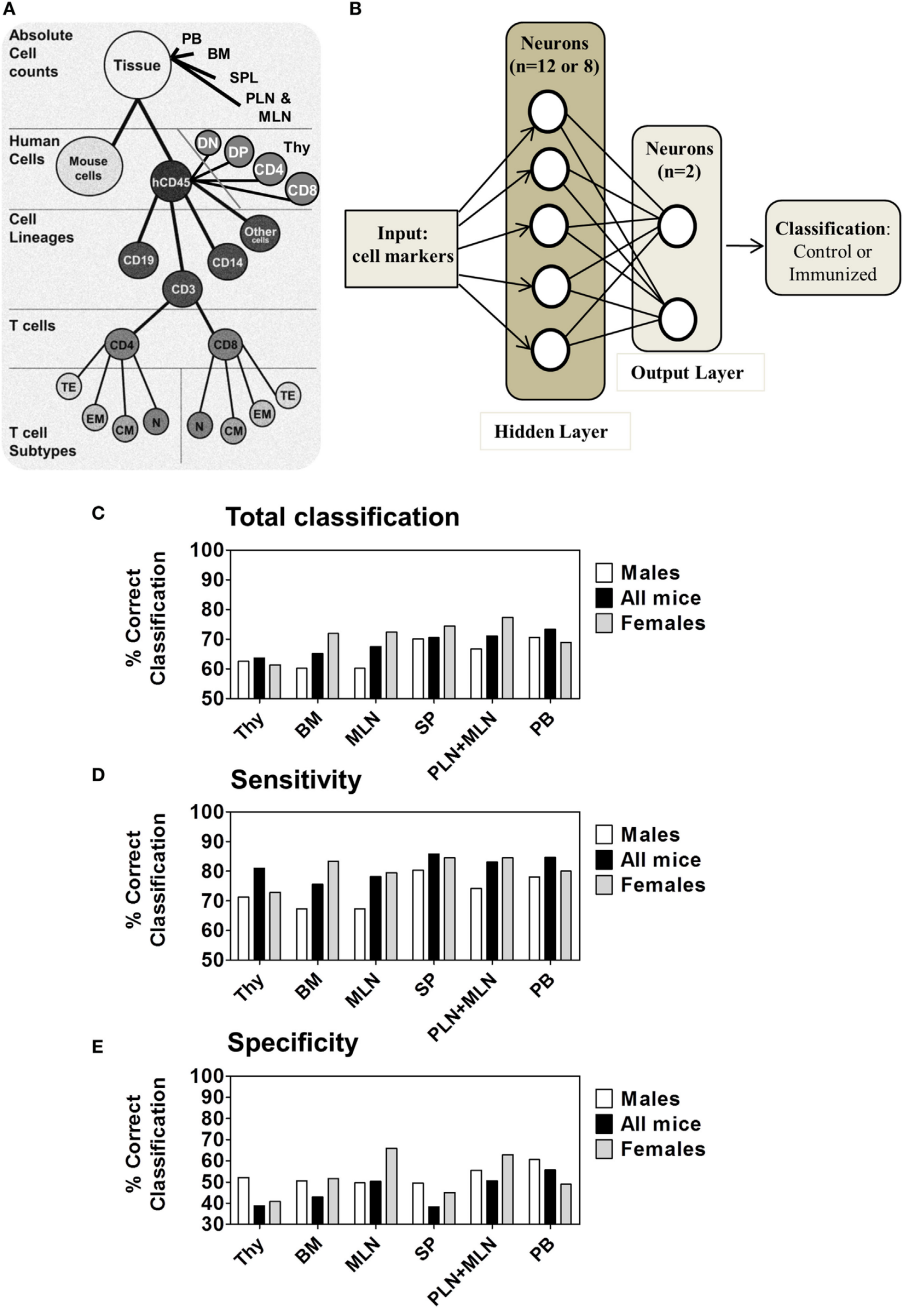
Multilayer Neural Network for performing classification. **(A)** Hierarchy of cell subsets used for analyses of primary and secondary lymphoid organs. **(B)** Schematic representation of the artificial neural network (ANN) structure used for samples classification containing an input module, a hidden layer and an output layer for classifying mice between control (*n* = 11) or immunized (*n* = 17) mice. **(C)** ANN total classification accuracy for distinct tissues by subject factor “immunization/control” among M mice (*n* = 14, white), all mice (*n* = 28, black) and F mice (*n* = 14, gray) analyzed for the 20 weeks model data set. **(D)** The sensitivity and **(E)** specificity measured for each gender and for all mice together. Tissues are presented in the order of increasing total classification accuracy when all mice were used.

We analyzed the dataset corresponding to the raw percentages of the measured human cell lineages in different tissues: BM, PB, Thy, SPL, PLN/MLN, and MLNs considering each tissue as an independent dataset. The scheme of the data hierarchy for the tissues is shown in Figure 7A. The markers from control (*n* = 11) and iDCpp65-immunized (*n* = 17) mice were used to feed 2000 ANN training-validation-test cycles per tissue. In each cycle, 70% of the samples were randomly selected and used for training, 15% of samples for validation during the training process, and 15% for testing. The output was the classification accuracy, i.e., the percentage of correct classifications (control group or iDCpp65 immunized) averaged over the 2000 ANN. Primary and secondary lymphoid tissues were ranked according to their potency to provide the correct output regarding the sample origin (Figure 7B). The classification of control versus iDCpp65-immunized mice for both genders was most efficient using data from PB (73.3% of all samples were classified correctly), followed by PLN merged with MLN (71.1%), and SPL (70.6%) (Figure 7C; Table S7 in Supplementary Material). The classification accuracy for Thy was the lowest among the tissues (Figure 7C). The above results depicted the heterogeneous impact of immunization in the different tissues from the perspective of the ability of ANN to distinguish control versus iDCpp65-immunized mice.

Subsequently, the mouse gender was taken into consideration. The same analysis was performed for female and male mice separately and results were provided in comparison with the full dataset of combined genders. The classification accuracy for female data was higher for all of the tissues in the combined genders analysis, with exception of analyses performed for Thy and PB. The highest classification accuracy per gender was detected in combined PLN and MLN of female mice (77.3%) (Figure 7C; Table S7 in Supplementary Material). Notably, classifications according to sensitivity (Figure 7D) were in general higher than specificity for all groups (Figure 7E), meaning that the ability to discriminate a mice belonging to the immunized group was higher than to classify a mice as belonging to the control group. For both classifications, the frequencies of correct classification for females were again superior compared with males, especially in combined PLN and MLN or MLN alone (F 84.5%/79.5% and M 74.2%/67.3%, respectively) (Table S7 in Supplementary Material). This gender-based classification supported the concept that lymphocyte markers of immunized female mice were more distinguishable than those of their male littermates.

### Correlation and Structural Elements of Immune-Phenotypic Markers in Tissues

In order to understand better the structural relationships between the measured markers in control and iDCpp65-immunized mice, we used a PCA approach. Our rationale was that these analyses might provide us with an estimate of which markers are the best predictors of immunization. Three PCAs were performed, either separately for each group (control or immunized), or including all mice (global PCA). Interestingly, the markers composing the first governing component of the global PCA (data not shown) also appeared in the separate PCAs (Table 1), but the group-specific PCA revealed more markers that are suitable to characterize intra-group heterogeneity.

**Table 1:** Immune-phenotypic markers measured in bone marrow (BM), Thy, SPL, PLN combined with MLN, MLN, and peripheral blood (PB) which are highly correlated (positive or negative correlation) with the first governing component of the principal component analysis performed in control and induced dendritic cells expressing pp65 (iDCpp65) mice.

Tissue	Control	iDCpp65
Thy	CD3%, CD4%, CD8%	CD3%, CD4%
	DP%	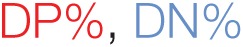
		DP#

BM	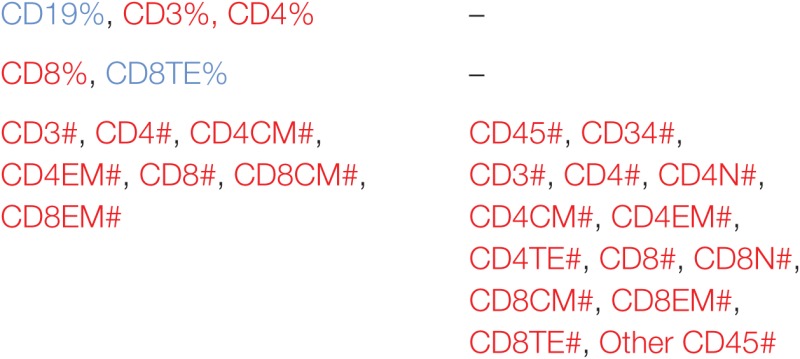	

SPL	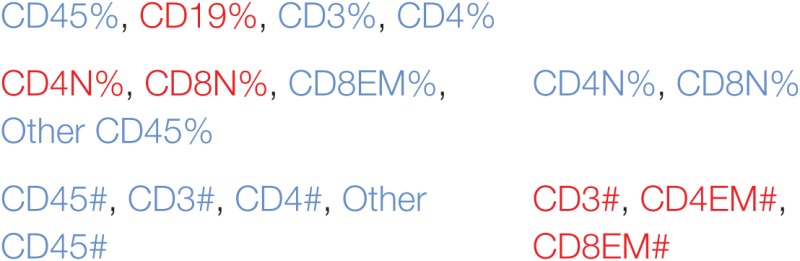	

PLN + mesenteric lymph node (MLN)	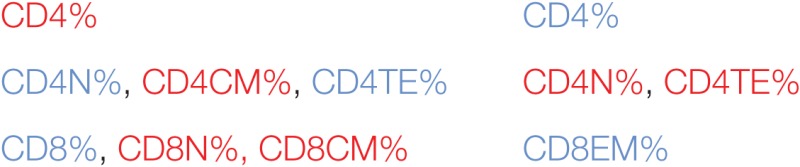	

MLN	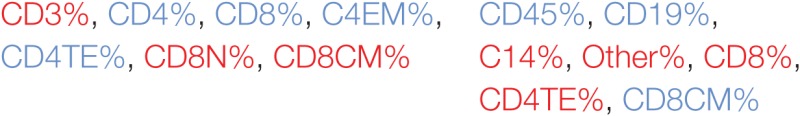	

PB	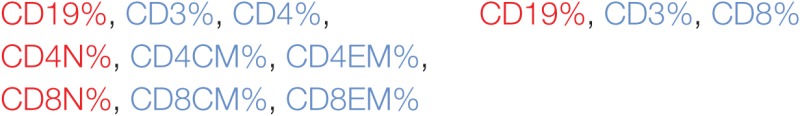	

*Color indicate the grade of correlation strength for different markers: red (correlation >80%) and blue (correlation <−80%), for frequencies (%), or absolute numbers (#). The number of samples (*n*) control/immunized = 11/17, respectively, except Thy (11/16), PLN + MLN (7/12), and MLN (10/11)*.

**Figure 8:**
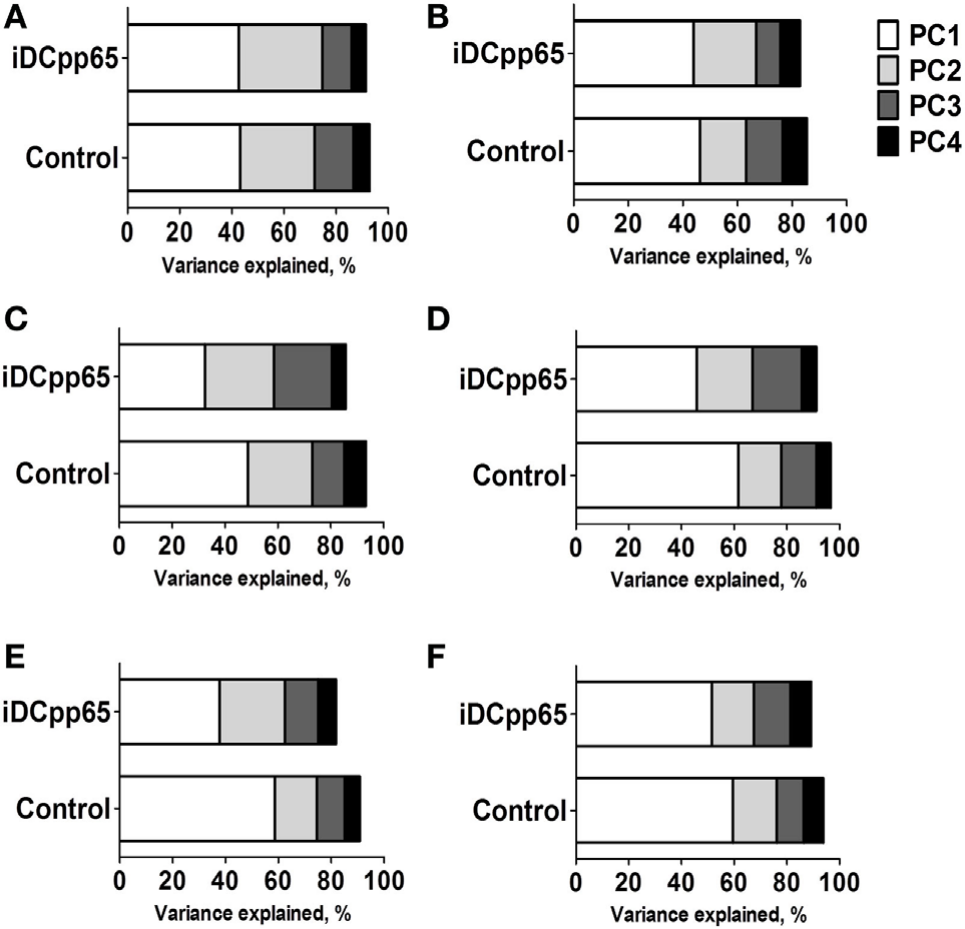
Principal components explaining the variance in different tissues. Variance explained by using the first four components in control and induced dendritic cells expressing pp65 (iDCpp65) mice. Principal components were established based on cell counts and frequencies in thymus, bone marrow (BM), and spleen. In the case of combined peripheral and mesenteric lymph nodes (MLN), peripheral blood (PB), and MLNs only frequencies were used. The analysis showed that the variance distributions among the first main components were similar for the two data sets. The analysis indicated that the variance distributions among the first main components were similar for thymus (Thy) **(A)** and BM **(B)** but more variable for spleen (SPL) **(C)**, combined peripheral and MLNs (PLN + MLN) **(D)**, PB **(E)**, and MLN **(F)**. The number of samples (*n*) for control/immunized varied for each tissue: thymus 11/16; PLN + MLN 7/12; MLN 10/11; SP, BM, PB 11/17.

**Figure 9:**
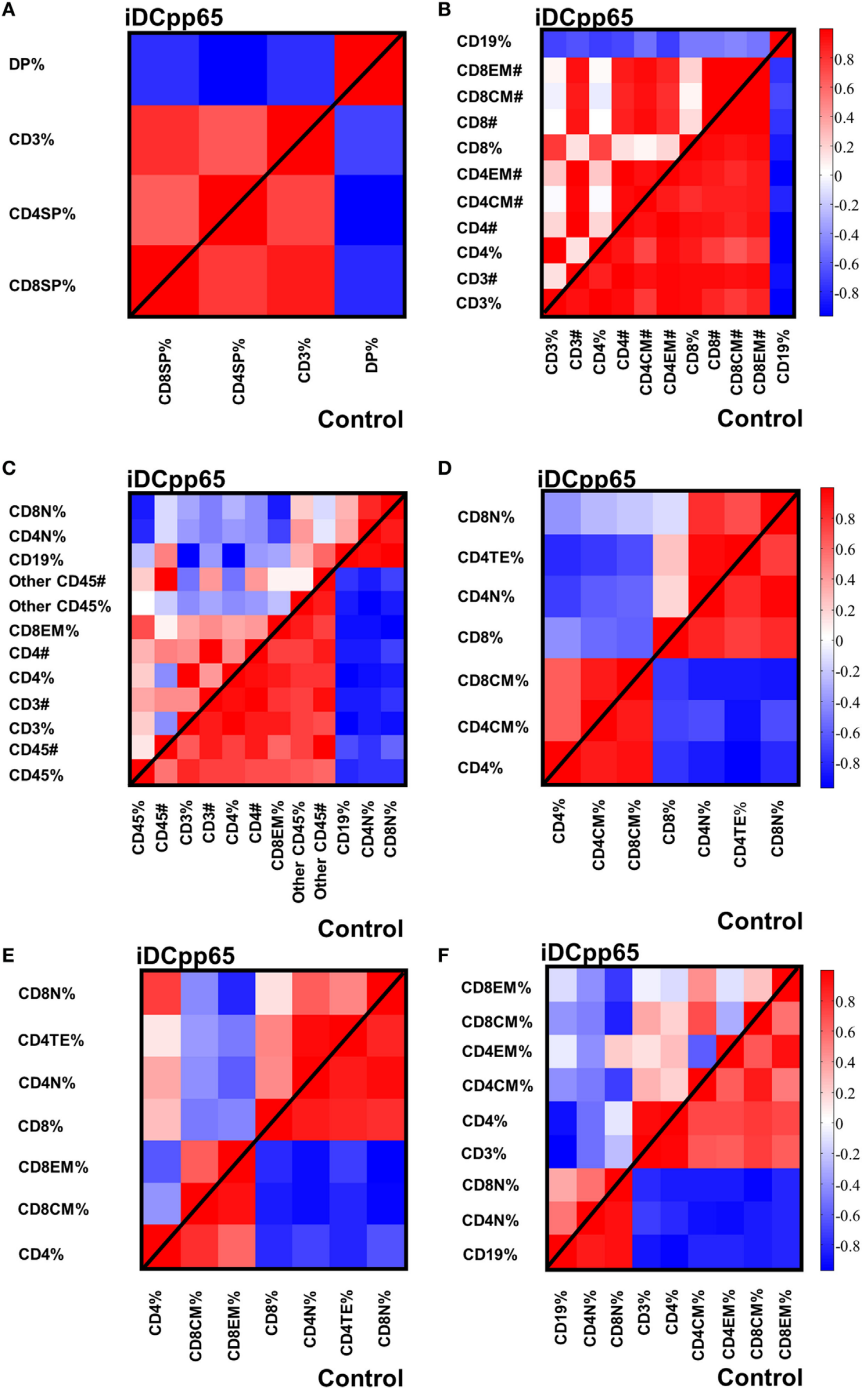
Heat-maps showing the correlations among specifically selected markers between control and induced dendritic cells expressing pp65 (iDCpp65) groups. The markers selected are the ones which highly contribute to the first governing component of the control mice in the different tissues. Analysis was performed using frequencies for bone marrow (BM), combined PLN and mesenteric lymph node (MLN), MLN, and peripheral blood (PB). In spleen (SPL) and thymus (Thy), principal component analysis was performed using cells counts and frequencies. The correlation maps between the specific markers in control and iDCpp65-immunized groups, determined in **(A)** thymus (Thy), **(B)** BM, **(C)** spleen (SPL), **(D)** combined peripheral and MLN (PLN + MLN), **(E)** MLN, and **(F)** PB. The number of samples (*n*) for control/immunized varied for each tissue: thymus 11/16; PLN + MLN 7/12; MLN 10/11; SP, BM, PB 11/17.

Initially, we tested how the variance of the data sets, obtained for the two mice groups in different tissues, could be distributed within the main components. In BM and Thy, PCA showed that the variance distributions among the first main components are similar in control and iDCpp65 mice (Figures 8A,B). We then proceeded to compare the correlation patterns between the two mice groups (Figures 9A,B; Figures S2A,B in Supplementary Material) by selecting the markers, which are highly correlated or anti-correlated with the first governing component of the control group (Table 1). These markers should contribute more to the total variance and thus to any dynamical changes among the control mice. The correlation patterns between control and iDCpp65 mice were similar in Thy (Figure 9A), meaning that the immunization did not impact the correlation between these markers. For instance, CD8SP% and CD3% cells were highly correlated in the control group, and this correlation was not altered by immunization. This result is in accordance with the classification accuracy performance where ANN could not provide a clear distinction between the control and iDCpp65 groups based on Thy specific marker analyses.

Interestingly, for BM, the correlation patterns between control and iDCpp65 mice showed differences in terms of the correlation strength that exists among specific markers (Figure 9B). For example, strongly positively correlated pairs of markers (CD3%, CD4CM#), (CD4%, CD8CM #), and (CD8%, CD8CM#) in the control group lost their correlation properties in the iDCpp65 group.

Noticeably, the same analysis in SPL revealed a considerable differentiation of the variance distributions in control and iDCpp65 mice (Figure 8C) and distinguishable correlation patterns in the heat-map analyses (Figure 9C; Figure S2C in Supplementary Material). An inversely correlated signature could be seen between CD45# and CD3% (positive correlation in control mice, equal to 0.6325, and negative correlation in iDCpp65 ones, equal to −0.4656) as well as between CD45# and CD4% (positive correlation in control mice, equal to 0.7179, and negative correlation in iDCpp65 ones, equal to −0.4493). The same inversely correlated signature could be also seen between CD45# and CD19% (negative correlation in control mice, equal to −0.6727, and positive correlation in iDCpp65 ones, equal to 0.5202). This indicated that the dynamics of cellular output in the spleen after iDCpp65 favored B cells and not T cells.

For combined PLN and MLN, the variance distributions and the correlative signatures between control and iDCpp65 mice showed considerable differences as well (Figures 8D and 9D; Figure S2D in Supplementary Material). More specifically, the positive strong correlation between CD8% and CD8N% in control mice (equal to 0.8345) became neutral in iDCpp65 (−0.1103). The same weakening in correlation strength was observed between CD8% and CD4N% (the correlation in control mice is equal to 0.8345, while in the immunized ones is equal to 0.1944). Thus, naïve T cell activation and loss of the naïve status by immunization, as determined by the PCA analysis, is physiologically meaningful.

The same analysis in MLN revealed important differences in the variance distribution in control and iDCpp65 mice (Figure 8E) and considerable correlation changes in the heat-map analysis (Figure 9E). Inverse correlated signatures could be seen between CD4% and CD8CM% (positive correlation in control mice, equal to 0.5969 and negative correlation in iDCpp65 ones, equal to −0.618), CD4% and CD8CM% (positive correlation in control mice, equal to 0.8102, and a weakly negative correlation in iDCpp65 ones, equal to −0.384). The same inversely correlated signature could be seen between CD4% and CD8N% (negative correlation in control mice, equal to −0.6346 and positive correlation in iDCpp65 ones, equal to 0.7513).

Finally, in PB, the same analysis revealed a striking difference in the variance distribution (Figure 8F) and considerable differences in the heat-map analysis (Figure 9F). Inversely correlated signatures could be seen between CD4CM% and CD4EM% (positive correlation in control mice, equal to 0.6414 and negative correlation in iDCpp65 ones, equal to −0.598) as well as between CD4EM% and CD8CM% (positive correlation in control mice, equal to 0.6567 and weakly negative correlation in iDCpp65 ones, equal to −0.3047). The positive strong correlation between CD4EM% and CD8EM% in control mice (equal to 0.94) became neutral in iDCpp65 (−0.09983) and the negative strong correlation between CD19% and CD4EM% in control mice (equal to −0.8088) becomes neutral in iDCpp65 (−0.06359).

The above results showed how we could exploit the intrinsic complexity and heterogeneity of the input markers to gain additional knowledge on the characteristic of an individual immunized mouse. More specifically, by checking the correlation patterns among specifically selected markers in an individual mouse, we could conclude about their immunization status. Together with the classification performance of ANN in the different tissues, these results predicted more pronounced effects of immunization in SPL and PLN combined with MLN compared with other tissues.

## Discussion

The use of immune deficient mice humanized with human HSCs to study and characterize the maturation of human T cells after immunizations in different lymphatic tissues generate large data sets and highly complex results. Long-term studies (20 or more weeks after transplantation) and large mouse cohorts (15 mice or more) have been commonly used (6). The initial HSC engraftment in BM, early T cell development in thymus and the egress of naïve T cells to the periphery recapitulate the general patterns found in immune competent mice and in humans (5, 33, 34) in the first 10–15 weeks after transplantation. However, analyses of the T cell maturation in secondary lymphoid organs have shown to be more heterogeneous and predictive of the quality of the immune reconstitution and development of mature T cells. This reflects the “personalized” condition of different CB donors with heterogeneous genetic backgrounds, which is amplified in a xenograft system. In addition, although the engraftment of human HSCs (35) and the higher thymic output in humanized female mice (25) had been previously reported, the relevance of the mouse sex in determining the impact of immunizations and the predictability of human T cell responses had not been presented. All these factors were taken in account when exploring humanized mice for testing a new vaccine type.

In the present work, we sought to evaluate the multidimensional spatial effects of a potent cellular vaccine against HCMV matched to the HSC donor and providing the three main relevant signals for both antigenic and homeostatic activation of T cells: an immune-dominant antigen presented *via* HLA class I and II, co-stimulatory ligands, and inflammatory cytokines. Thus, iDCpp65 which are viable for 2–3 weeks *in vivo* and effectively migrate to lymph node structures (19) were used to accelerate and potently boost the human T cell development and functional responses in humanized mice. As anticipated, improved human T cell development and maturation were longitudinally observed in PB and terminally in several lymphatic tissues in iDCpp65-immunized mice at 20-weeks after HSCT. These results complemented previous findings obtained in shorter (16 weeks) and in longer (up to 36 weeks) iDCpp65 immunization models (20, 24). Advanced statistical analyses showed that iDCpp65 immunizations promoted a typical memory T cell signature (above all for CD8^+^ T cells) which was most prominent for T cells homing lymph nodes and spleen. As most of the studies using humanized mice have focused on analyses of human cells in blood and spleen (5, 8, 18) it is important to emphasize that, as seen from the ANN and PCA analyses, the quantity and quality of human T cell reactivity in peripheral and MLNs (even if they are small and difficult to be sampled) have to be taken in account, as lymph nodes represent the prime tissue for interactions between antigen-presenting cells with naïve CD8^+^ and CD4^+^ T cells. Further, the levels of human IFN-γ in plasma increased upon immunization.

In general, both cellular and cytokine immune effects were more accentuated for female mice. This confirmed and expanded our previously reported observation that humanized female mice have higher output of naïve T cells than humanized male mice until 12 weeks after HSCT (25). Around 16 weeks after HSCT, male mice showed higher development of mature T cells and by 20 weeks after HSCT, the frequencies of human CD45^+^ cells, naïve and memory T cells in PB equalized between the sexes. Notta et al. showed that between 10 and 12 weeks after HSCT, females transplanted with limiting amounts of HSCs obtained from several CB units generally exhibited a higher frequency of huCD45^+^ cells than male mice (35). Therefore, CB-HSCT in humanized mice could potentially mirror the effect of sex steroids on human immune reconstitution since temporarily blocking sex steroids before HSCT in patients, increased thymus function and enhanced the rate of T-cell regeneration (36). Responses to various types of vaccination are often higher among women [for a review see Ref. (37)], who are able to mount stronger humoral responses than men. A possible explanation for this phenomenon is based again on major sex steroid hormones such as the typical “female” hormone estradiol that enhances the adaptive and innate immune systems, and the “male” hormone testosterone considered immune suppressive. Noteworthy, women display higher T helper type 2 (Th2) responses, whereas males favor Th1 responses (37). Although sex-specific responses to distinct vaccines are not usually considered and have been reported in a few clinical trials, this is an important factor also to be considered in preclinical research, when testing new vaccine types, including when humanized mice are used as a potency model. In the current model, we obtained not only higher responses, but exploring the ANN, also a better prediction of response. Thus, as a logical approach to reduce the numbers of humanized mice when testing a vaccine is initially favoring the use of female mice. In addition, from now on, studies on humanized mice should consider male and female responses as distinct responses, and should be analyzed separately and compared.

We also showed that the statistical methods can be complemented with an ANN algorithm in order to pin down the complexity of a multidimensional data sets including usual immune markers such as frequencies of the human cell phenotypes among lymphatic tissues and considering mouse sexes. As generally proposed for ANNs (38), we were able to demonstrate here that the ANN based on the humanized mouse data could “learn” to recognize the immune properties of immunized versus control mice. For studies in humans, ANNs were built with independent immunologic variables such as cell proliferation, phenotypic markers, and cytokine expression in the context of prostate cancer and in HSCT patients (39–41). To our knowledge, the application of ANNs to humanized mouse models for predicting the accuracy of immune response or defining signatures of T cell responses was not previously performed. The identification of lymph node and spleen as the most predictive organs for the immune state of control versus immunized mice might be further improved by releasing the assumption of statistically independent tissues. Along with local immune population dynamics, it would be of interest to investigate the immune cell trafficking dynamics between different tissues (42, 43). In this way, we could relax the assumption of tissue independence. However, the immune cell trafficking is an open challenge yet to be solved in future research.

Altogether, the current approach, modalities of analyses and observations give valuable information for further planning of *in vivo* testing of vaccines and immune modulators in humanized mice. The 3R principle (*R*eplace animal testing, *R*educe the number of animals, and *R*efine the analyses) can thus be advanced for Reduce and Refine: (i) by using (at least initially) female mice and (ii) exploring bio-informatics methods such as ANN to complement traditional statistical analyses in order to define the most important tissues (such as spleen and lymph nodes) and the PCA that reveal signatures and correlations of immune responses for different lymphatic tissues.

## Ethics Statement

All subjects donating cord blood provided written informed consent. This study was approved by the Ethics Committee of Hannover Medical School.

## Author Contributions

RS planed the project, designed experiments, obtained funding and regulatory approvals, enrolled collaborators, interpreted the data, and wrote and edited the manuscript. VV conducted experiments, analyzed data, and wrote the first manuscript draft. BS, ST, AS, LG, and CR assisted in preparation and analyses of humanized mice. CF performed the human cytokine array analyses. CK assisted in the procurement and collection of HSC for the studies. LS performed the statistical analyses. AR, PR, HH, and MM-H performed the ANN and PCA analyses, interpreted the data, and wrote and edited the manuscript. SK and UK assisted in the execution of the iDCpp65 quality control analyses, and revised the manuscript.

## Conflict of Interest Statement

One of the corresponding authors is currently applying for a patent related to the content of the manuscript: R. Stripecke, G. Salguero, A. Daenthasanmak, A. Ganser. “Induced dendritic cells and uses thereof” (PCT/EP2013/052485). All other authors declare that the research was conducted in the absence of any commercial or financial relationships that could be construed as a potential conflict of interest.

## Abbreviations

aAPC, artificial antigen-presenting cell; Ab, Antibody; ANN, artificial neural network; ANOVA, analysis of variance; B. D., below detection; BM, bone marrow; BLT, bone marrow, liver, thymus (mouse model); CB, cord blood; CM, central memory; CTL, cytotoxic lymphocytes; DN, double negative; DP, double positive; DC, dendritic cell; ELISPOT, enzyme-linked immuno spot assay; EM, effector memory; FBS, fetal bovine serum; HCMV, human cytomegalovirus; HIV, human immunodeficiency virus; HLA, human leukocyte antigen; HSCs, hematopoietic stem cells; HSCT, hematopoietic stem cell transplantation; iDCpp65, induced dendritic cells expressing pp65; IDLV-G2a-pp65, integration-deficient lentiviral vector co-expressing GM-CSF/IFN-α and the HCMV-pp65; IL, interleukin; MCP-1, monocyte chemoattractant protein 1; MLNs, mesenteric lymph nodes; N, naïve; NRG, NOD.Cg-Rag1*^tm1Mom^*-Il2rγ*^tm1Wj^;* PB, peripheral blood; PBS, phosphate-buffered saline; PCA, principal component analysis; Pp65, phosphoprotein 65; PLN, peripheral lymph nodes; SPL, spleen; TCRαβ, T cell receptor αβ; TE, terminal effector; Th1, T helper type 1; Thy, thymus; WT1, Wilms tumor 1 protein.
