# Supplementary material for: Multidimensional Analysis Integrating Human T-Cell Signatures in Lymphatic Tissues with Sex of Humanized Mice for Prediction of Responses after Dendritic Cell Immunization
Source: Front Immunol. 2017 Dec 8;8:1709. doi: 10.3389/fimmu.2017.01709 (PMC5727047; doi:10.3389/fimmu.2017.01709)
Supplement: Supplementary file 1 [file Table_1.docx]

**Supplementary Table 1. Least squares means estimation of mean relative frequency for the analysis of peripheral blood data at week 20 post HSC transplantation.**

|  | **Female, n=14** | | **Male, n=14** | | **Group, n=28** | |
| --- | --- | --- | --- | --- | --- | --- |
|  | **iDCpp65, n=9** | **Control, n=5** | **iDCpp65, n=8** | **Control, n=6** | **iDCpp65, n=17** | **Control, n=11** |
| **CD19** |  | | | |  | |
| **LSM** | 42.78 | 37.97 | 27.87 | 36.44 | 35.57 | 37.35 |
| **OR** | 1.22 | | 0.67 | | 0.93 | |
| **p-value^1^** | 0.58 | | 0.28 | | 0.77 | |
|  |  | | | | | |
| **Other CD45^+^** |  | |  | |  | |
| **LSM** | 13.21 | 16.96 | 13.70 | 22.53 | 13.52 | 19.89 |
| **OR** | 0.74 | | 0.55 | | 0.63 | |
| **p-value** | 0.36 | | ***0.04*** | | ***0.04*** | |
|  |  | | | | | |
| **CD3** |  | |  | |  | |
| **LSM** | 44.28 | 40.12 | 60.04 | 39.13 | 51.73 | 39.77 |
| **OR** | 1.19 | | 2.34 | | 1.62 | |
| **p-value** | 0.67 | | ***0.04*** | | 0.10 | |
|  |  | | | | | |
| **CD4** |  | |  | |  | |
| **LSM** | 29.37 | 24.35 | 42.48 | 29.08 | 35.42 | 27.17 |
| **OR** | 1.29 | | 1.80 | | 1.47 | |
| **p-value** | 0.48 | | 0.09 | | 0.14 | |
|  |  | | | | | |
| **CD4N** |  | |  | |  | |
| **LSM** | 19.85 | 26.08 | 15.08 | 19.29 | 17.62 | 22.30 |
| **OR** | 0.70 | | 0.74 | | 0.74 | |
| **p-value** | 0.46 | | 0.55 | | 0.40 | |
|  |  | | | | | |
| **CD4CM** |  | |  | |  | |
| **LSM** | 49.13 | 45.38 | 58.94 | 49.67 | 53.76 | 47.73 |
| **OR** | 1.16 | | 1.45 | | 1.27 | |
| **p-value** | 0.57 | | 0.15 | | 0.21 | |
|  |  | | | | | |
| **CD4EM** |  | |  | |  | |
| **LSM** | 32.38 | 22.52 | 29.23 | 27.90 | 30.92 | 25.40 |
| **OR** | 1.65 | | 1.07 | | 1.31 | |
| **p-value** | 0.12 | | 0.83 | | 0.22 | |
|  |  | | | | | |
| **CD4TE** |  | |  | |  | |
| **LSM** | 0.76 | 0.79 | 0.42 | 0.54 | 0.55 | 0.63 |
| **OR** | 0.96 | | 0.78 | | 0.87 | |
| **p-value** | 0.92 | | 0.50 | | 0.62 | |
|  |  | | | | | |
| **CD8** |  | |  | |  | |
| **LSM** | 13.06 | 11.45 | 15.91 | 8.09 | 14.43 | 9.57 |
| **OR** | 1.16 | | 2.15 | | 1.59 | |
| **p-value** | 0.58 | | ***0.01*** | | ***0.03*** | |
|  |  | | | | | |
| **CD8N** |  | |  | |  | |
| **LSM** | 39.39 | 54.86 | 33.33 | 34.75 | 36.80 | 43.66 |
| **OR** | 0.53 | | 0.94 | | 0.75 | |
| **p-value** | 0.27 | | 0.91 | | 0.48 | |
|  |  | | | | | |
| **CD8CM** |  | |  | |  | |
| **LSM** | 41.29 | 27.67 | 40.38 | 39.64 | 40.93 | 33.97 |
| **OR** | 1.84 | | 1.03 | | 1.35 | |
| **p-value** | 0.16 | | 0.94 | | 0.31 | |
|  |  | | | | | |
| **CD8EM** |  | |  | |  | |
| **LSM** | 21.95 | 16.15 | 25.41 | 16.68 | 23.56 | 16.51 |
| **OR** | 1.46 | | 1.70 | | 1.56 | |
| **p-value** | 0.39 | | 0.17 | | 0.13 | |
|  |  | | | | | |
| **CD8TE** |  | |  | |  | |
| **LSM** | 2.86 | 2.12 | 2.70 | 1.45 | 2.79 | 1.62 |
| **OR** | 1.36 | | 1.89 | | 1.75 | |
| **p-value** | 0.56 | | 0.14 | | 0.10 | |

Note: LSM: least squares means estimation; OR: odds ratio (between iDCpp65 and control per gender; between iDCpp65 and control irrespective of gender).

^1^P-value less than 0.05 is indicated by black and italic.
