# Supplementary material for: Multidimensional Analysis Integrating Human T-Cell Signatures in Lymphatic Tissues with Sex of Humanized Mice for Prediction of Responses after Dendritic Cell Immunization
Source: Front Immunol. 2017 Dec 8;8:1709. doi: 10.3389/fimmu.2017.01709 (PMC5727047; doi:10.3389/fimmu.2017.01709)
Supplement: Supplementary file 2 [file Table_2.docx]

**Supplementary Table 2. Least squares means estimation of mean pg/ml for the analysis of human cytokines in mice plasma.**

|  | **Female, n=14** | | **Male, n=14** | | **Group, n=28** | |
| --- | --- | --- | --- | --- | --- | --- |
|  | **iDCpp65 n=9** | **Control n=5** | **iDCpp65 n=8** | **Control n=6** | **iDCpp65 n=17** | **Control n=11** |
| **GM-CSF** |  | | | |  | |
| **LSM** | 7.23 | 3.15 | 56.30 | 22.10 | 31.76 | 11.57 |
| **RR** | 2.30 | | 2.55 | | 2.74 | |
| **p-value^1^** | 0.057 | | 0.18 | | 0.15 | |
|  |  | | | | | |
| **TNFα** |  | |  | |  | |
| **LSM** | 5.06 | 6.01 | 9.26 | 9.09 | 7.16 | 7.69 |
| **RR** | 0.84 | | 1.02 | | 0.93 | |
| **p-value** | 0.73 | | 0.97 | | 0.85 | |
|  |  | | | | | |
| **IFN-γ** |  | |  | |  | |
| **LSM** | 23.80 | 3.83 | 58.97 | 26.68 | 41.39 | 13.98 |
| **RR** | 6.22 | | 2.21 | | 2.96 | |
| **p-value** | ***0.003*** | | 0.28 | | 0.092 | |

Note: LSM: least squares means estimation; RR: rate ratio (between iDCpp65 and control per gender; between iDCpp65 and control irrespective of gender).

^1^P-value less than 0.05 is indicated by black and italic.
