# Supplementary material for: Multidimensional Analysis Integrating Human T-Cell Signatures in Lymphatic Tissues with Sex of Humanized Mice for Prediction of Responses after Dendritic Cell Immunization
Source: Front Immunol. 2017 Dec 8;8:1709. doi: 10.3389/fimmu.2017.01709 (PMC5727047; doi:10.3389/fimmu.2017.01709)
Supplement: Supplementary file 3 [file Table_3.docx]

**Supplementary Table 3. Least squares means estimation of mean relative frequency and counts for the analysis of Bone marrow data.**

|  | **Bone marrow, %** | | | | | | | **Bone marrow, #** | | | | | |
| --- | --- | --- | --- | --- | --- | --- | --- | --- | --- | --- | --- | --- | --- |
|  | **Female. n=14** | | **Male, n=14** | | **Group** | | | **Female, n=14** | | **Male, n=14** | | **Group** | |
|  | **iDCpp65**  **n=9** | **Control**  **n=5** | **iDCpp65**  **n=8** | **Control**  **n=6** | **iDCpp65 n=17** | **Control n=11** | | **iDCpp65 n=9** | **Control n=5** | **iDCpp65 n=8** | **Control n=6** | **iDCpp65 n=17** | **Control n=11** |
| **CD19** |  | | | | | | |  | | | | | |
| **LSM** | 53.82 | 32.09 | 40.17 | 36.17 | 47.36 | 34.51 | | 192325 | 366799 | 303622 | 376871 | 244700 | 372293 |
| **OR/RR** | 2.47 | | 1.18 | | 1.71 | | | 0.52 | | 0.81 | | 0.66 | |
| **p-value^1^** | 0.058 | | 0.70 | | 0.11 | | | 0.17 | | 0.43 | | 0.11 | |
|  |  | | | | | | |  | | | | | |
| **CD34** |  | |  | |  | | |  | |  | |  | |
| **LSM** | 5.77 | 4.44 | 7.41 | 9.16 | 6.69 | | 6.78 | 23353 | 47760 | 64305 | 100626 | 42624 | 76596 |
| **OR/RR** | 1.32 | | 0.79 | | 0.98 | | | 0.49 | | 0.64 | | 0.56 | |
| **p-value** | 0.31 | | 0.27 | | 0.94 | | | 0.15 | | 0.12 | | ***0.048*** | |
|  |  | | | | | | |  | | | | | |
| **CD3** |  | |  | |  | | |  | |  | |  | |
| **LSM** | 16.46 | 14.80 | 18.44 | 14.65 | 17.37 | | 14.74 | 17845 | 218105 | 100455 | 84742 | 56721 | 145362 |
| **OR/RR** | 1.13 | | 1.32 | | 1.22 | | | 0.08 | | 1.18 | | 0.39 | |
| **p-value** | 0.83 | | 0.62 | | 0.63 | | | ***0.0001*** | | 0.72 | | 0.057 | |
|  |  | | | | | | |  | | | | | |
| **CD4** |  | |  | |  | | |  | |  | |  | |
| **LSM** | 13.37 | 11.38 | 15.64 | 12.43 | 14.42 | | 11.97 | 14856 | 161863 | 83378 | 69754 | 47102 | 111622 |
| **OR/RR** | 1.20 | | 1.31 | | 1.24 | | | 0.09 | | 1.19 | | 0.42 | |
| **p-value** | 0.75 | | 0.63 | | 0.59 | | | ***0.0001*** | | 0.71 | | 0.068 | |
|  |  | | | | | | |  | | | | | |
| **CD4N** |  | |  | |  | | |  | |  | |  | |
| **LSM** | 0.95 | 1.58 | 0.97 | 1.25 | 0.96 | | 1.39 | 58 | 607 | 389 | 332 | 214 | 457 |
| **OR/RR** | 0.59 | | 0.78 | | 0.69 | | | 0.09 | | 1.17 | | 0.47 | |
| **p-value** | 0.28 | | 0.60 | | 0.27 | | | ***0.0006*** | | 0.71 | | 0.10 | |
|  |  | | | | | | |  | | | | | |
| **CD4CM** |  | |  | |  | | |  | |  | |  | |
| **LSM** | 9.48 | 18.13 | 19.05 | 20.52 | 13.68 | | 19.81 | 1036 | 25705 | 15797 | 15620 | 7982 | 20205 |
| **OR/RR** | 0.47 | | 0.91 | | 0.64 | | | 0.04 | | 1.01 | | 0.39 | |
| **p-value** | ***0.01*** | | 0.69 | | ***0.04*** | | | ***0.0001*** | | 0.98 | | 0.07 | |
|  |  | | | | | | |  | | | | | |
| **CD4EM** |  | |  | |  | | |  | |  | |  | |
| **LSM** | 87.35 | 75.55 | 79.55 | 77.06 | 83.85 | | 76.18 | 13568 | 134705 | 66521 | 53303 | 38487 | 90304 |
| **OR/RR** | 2.24 | | 1.16 | | 1.62 | | | 0.10 | | 1.25 | | 0.43 | |
| **p-value** | ***0.006*** | | 0.55 | | ***0.02*** | | | ***0.0003*** | | 0.63 | | 0.07 | |
|  |  | | | | | | |  | | | | | |
| **CD4TE** |  | |  | |  | | |  | |  | |  | |
| **LSM** | 2.42 | 2.43 | 1.54 | 2.01 | 1.98 | | 2.24 | 195 | 774 | 668.30 | 528 | 418 | 639 |
| **OR/RR** | 0.99 | | 0.76 | | 0.88 | | | 0.25 | | 1.27 | | 0.65 | |
| **p-value** | 0.99 | | 0.55 | | 0.70 | | | ***0.009*** | | 0.56 | | 0.25 | |
|  |  | | | | | | |  | | | | | |
| **CD8** |  | |  | |  | | |  | |  | |  | |
| **LSM** | 2.08 | 1.94 | 2.20 | 1.69 | 2.14 | | 1.80 | 2360 | 36012 | 13295 | 10331 | 7506 | 22004 |
| **OR/RR** | 1.07 | | 1.31 | | 1.19 | | | 0.06 | | 1.28 | | 0.34 | |
| **p-value** | 0.88 | | 0.57 | | 0.60 | | | ***0.0001*** | | 0.63 | | 0.07 | |
|  |  | | | | | | |  | | | | | |
| **CD8N** |  | |  | |  | | |  | |  | |  | |
| **LSM** | 2.95 | 2.94 | 2.27 | 3.18 | 2.55 | | 3.10 | 42 | 500 | 219 | 220 | 125 | 347 |
| **OR/RR** | 1.00 | | 0.71 | | 0.82 | | | 0.08 | | 0.99 | | 0.36 | |
| **p-value** | 0.99 | | 0.37 | | 0.50 | | | ***0.0001*** | | 0.99 | | 0.07 | |
|  |  | | | | | | |  | | | | | |
| **CD8CM** |  | |  | |  | | |  | |  | |  | |
| **LSM** | 7.03 | 13.81 | 11.33 | 12.75 | 9.23 | | 13.30 | 132 | 5103 | 1677 | 1333 | 859 | 3047 |
| **OR/RR** | 0.47 | | 0.87 | | 0.66 | | | 0.03 | | 1.26 | | 0.28 | |
| **p-value** | ***0.004*** | | 0.48 | | ***0.02*** | | | ***0.0001*** | | 0.69 | | 0.07 | |
|  |  | | | | | | |  | | | | | |
| **CD8EM** |  | |  | |  | | |  | |  | |  | |
| **LSM** | 78.02 | 62.31 | 75.51 | 68.04 | 76.81 | | 65.47 | 1921 | 28723 | 10121 | 7599 | 5780 | 17201 |
| **OR/RR** | 2.15 | | 1.45 | | 1.75 | | | 0.07 | | 1.33 | | 0.34 | |
| **p-value** | 0.06 | | 0.34 | | 0.053 | | | ***0.0001*** | | 0.59 | | 0.07 | |
|  |  | | | | | | |  | | | | | |
| **CD8TE** |  | |  | |  | | |  | |  | |  | |
| **LSM** | 16.12 | 19.89 | 13.06 | 17.10 | 14.65 | | 18.38 | 265 | 1691 | 1280 | 1179 | 743 | 1412 |
| **OR/RR** | 0.77 | | 0.73 | | 0.76 | | | 0.16 | | 1.08 | | 0.53 | |
| **p-value** | 0.61 | | 0.53 | | 0.45 | | | ***0.0002*** | | 0.92 | | 0.28 | |

Note: LSM: least squares means estimation; OR: odds ratio (between iDCpp65 and control per gender; between iDCpp65 and control irrespective of gender); RR: rate ratio (between iDCpp65 and control per gender; between iDCpp65 and control irrespective of gender).

^1^P-value less than 0.05 is indicated by black and italic.
