# Supplementary material for: Multidimensional Analysis Integrating Human T-Cell Signatures in Lymphatic Tissues with Sex of Humanized Mice for Prediction of Responses after Dendritic Cell Immunization
Source: Front Immunol. 2017 Dec 8;8:1709. doi: 10.3389/fimmu.2017.01709 (PMC5727047; doi:10.3389/fimmu.2017.01709)
Supplement: Supplementary file 4 [file Table_4.docx]

**Supplementary Table 4. Least squares means estimation of mean relative frequency and counts for the analysis of Spleen data.**

|  | **Spleen, %** | | | | | | | **Spleen, #** | | | | | |
| --- | --- | --- | --- | --- | --- | --- | --- | --- | --- | --- | --- | --- | --- |
|  | **Female. n=14** | | **Male, n=14** | | **Group** | | | **Female, n=14** | | **Male, n=14** | | **Group** | |
|  | **iDCpp65**  **n=9** | **Control**  **n=5** | **iDCpp65**  **n=8** | **Control**  **n=6** | **iDCpp65 n=17** | **Control n=11** | | **iDCpp65 n=9** | **Control n=5** | **iDCpp65 n=8** | **Control n=6** | **iDCpp65 n=17** | **Control n=11** |
| **CD19** |  | | | | | | |  | | | | | |
| **LSM** | 61.55 | 59.61 | 48.12 | 59.28 | 55.28 | 59.31 | | 7456895 | 5414151 | 5096444 | 7405974 | 6346094 | 6500600 |
| **OR/RR** | 1.08 | | 0.64 | | 0.85 | | | 1.38 | | 0.69 | | 0.98 | |
| **p-value^1^** | 0.83 | | 0.23 | | 0.55 | | | 0.25 | | 0.32 | | 0.92 | |
|  |  | | | | | | |  | | | | | |
| **Other CD45^+^** |  | |  | |  | | |  | |  | |  | |
| **LSM** | 12.84 | 13.62 | 11.97 | 14.61 | 12.43 | | 14.16 | 1360410 | 1613683 | 1117422 | 1929141 | 1246063 | 1785751 |
| **OR/RR** | 0.93 | | 0.79 | | 0.86 | | | 0.84 | | 0.58 | | 0.70 | |
| **p-value** | 0.69 | | 0.18 | | 0.22 | | | 0.63 | | 0.09 | | 0.14 | |
|  |  | | | | | | |  | | | | | |
| **CD3** |  | |  | |  | | |  | |  | |  | |
| **LSM** | 27.56 | 24.51 | 41.42 | 27.25 | 33.89 | | 26.36 | 2305483 | 3899794 | 3575212 | 3477614 | 2903002 | 3669514 |
| **OR/RR** | 1.17 | | 1.89 | | 1.43 | | | 0.59 | | 1.03 | | 0.79 | |
| **p-value** | 0.73 | | 0.14 | | 0.26 | | | 0.28 | | 0.94 | | 0.47 | |
|  |  | | | | | | |  | | | | | |
| **CD4** |  | |  | |  | | |  | |  | |  | |
| **LSM** | 18.90 | 15.96 | 29.93 | 19.21 | 23.87 | | 18.07 | 1494614 | 3187952 | 2512764 | 2472777 | 1973743 | 2797856 |
| **OR/RR** | 1.23 | | 1.80 | | 1.42 | | | 0.47 | | 1.02 | | 0.70 | |
| **p-value** | 0.65 | | 0.16 | | 0.26 | | | 0.15 | | 0.97 | | 0.32 | |
|  |  | | | | | | |  | | | | | |
| **CD4N** |  | |  | |  | | |  | |  | |  | |
| **LSM** | 9.70 | 18.53 | 9.96 | 17.06 | 9.83 | | 17.72 | 118835 | 185931 | 137479 | 191535 | 127609 | 188987 |
| **OR/RR** | 0.47 | | 0.54 | | 0.51 | | | 0.64 | | 0.72 | | 0.67 | |
| **p-value** | 0.14 | | 0.21 | | 0.06 | | | 0.32 | | 0.33 | | 0.15 | |
|  |  | | | | | | |  | | | | | |
| **CD4CM** |  | |  | |  | | |  | |  | |  | |
| **LSM** | 13.70 | 20.89 | 32.72 | 35.83 | 22.20 | | 29.37 | 229763 | 392252 | 836952 | 962772 | 515499 | 703444 |
| **OR/RR** | 0.60 | | 0.87 | | 0.69 | | | 0.58 | | 0.87 | | 0.73 | |
| **p-value** | 0.20 | | 0.67 | | 0.21 | | | 0.29 | | 0.78 | | 0.47 | |
|  |  | | | | | | |  | | | | | |
| **CD4EM** |  | |  | |  | | |  | |  | |  | |
| **LSM** | 68.61 | 46.27 | 56.14 | 45.65 | 62.88 | | 45.98 | 1052829 | 2394631 | 1489787 | 1227583 | 1258455 | 1758059 |
| **OR/RR** | 2.54 | | 1.52 | | 1.99 | | | 0.44 | | 1.21 | | 0.72 | |
| **p-value** | 0.06 | | 0.33 | | ***0.04*** | | | 0.18 | | 0.68 | | 0.44 | |
|  |  | | | | | | |  | | | | | |
| **CD4TE** |  | |  | |  | | |  | |  | |  | |
| **LSM** | 6.49 | 8.59 | 2.87 | 5.42 | 4.68 | | 7.00 | 92821 | 215744 | 49447 | 90351 | 72409 | 147347 |
| **OR/RR** | 0.74 | | 0.52 | | 0.65 | | | 0.43 | | 0.55 | | 0.49 | |
| **p-value** | 0.29 | | 0.07 | | 0.10 | | | 0.11 | | 0.07 | | 0.07 | |
|  |  | | | | | | |  | | | | | |
| **CD8** |  | |  | |  | | |  | |  | |  | |
| **LSM** | 7.87 | 2.31 | 10.15 | 6.61 | 9.18 | | 4.24 | 689697 | 245784 | 871204 | 714845 | 775112 | 501635 |
| **OR/RR** | 3.60 | | 1.60 | | 2.28 | | | 2.81 | | 1.22 | | 1.54 | |
| **p-value** | ***0.01*** | | 0.14 | | ***0.01*** | | | ***0.03*** | | 0.54 | | 0.15 | |
|  |  | | | | | | |  | | | | | |
| **CD8N** |  | |  | |  | | |  | |  | |  | |
| **LSM** | 18.69 | 39.05 | 22.62 | 29.36 | 20.63 | | 33.64 | 104738 | 130620 | 129884 | 132104 | 116571 | 131429 |
| **OR/RR** | 0.39 | | 0.70 | | 0.51 | | | 0.80 | | 0.98 | | 0.89 | |
| **p-value** | 0.06 | | 0.48 | | 0.07 | | | 0.72 | | 0.94 | | 0.70 | |
|  |  | | | | | | |  | | | | | |
| **CD8CM** |  | |  | |  | | |  | |  | |  | |
| **LSM** | 7.57 | 16.79 | 22.11 | 18.97 | 13.58 | | 19.00 | 50531 | 37236 | 229468 | 143896 | 134736 | 95414 |
| **OR/RR** | 0.41 | | 1.21 | | 0.67 | | | 1.36 | | 1.59 | | 1.41 | |
| **p-value** | ***0.02*** | | 0.53 | | 0.16 | | | 0.55 | | 0.30 | | 0.44 | |
|  |  | | | | | | |  | | | | | |
| **CD8EM** |  | |  | |  | | |  | |  | |  | |
| **LSM** | 45.69 | 28.81 | 46.26 | 33.67 | 45.96 | | 31.42 | 338486 | 48304 | 446803 | 312172 | 389458 | 192231 |
| **OR/RR** | 2.08 | | 1.69 | | 1.86 | | | 7.01 | | 1.43 | | 2.03 | |
| **p-value** | 0.11 | | 0.22 | | 0.06 | | | ***0.001*** | | 0.46 | | 0.13 | |
|  |  | | | | | | |  | | | | | |
| **CD8TE** |  | |  | |  | | |  | |  | |  | |
| **LSM** | 28.92 | 14.55 | 9.59 | 17.55 | 18.83 | | 17.61 | 195921 | 29680 | 65059 | 126457 | 134338 | 82467 |
| **OR/RR** | 2.39 | | 0.50 | | 1.09 | | | 6.60 | | 0.51 | | 1.63 | |
| **p-value** | ***0.01*** | | ***0.04*** | | 0.77 | | | ***0.001*** | | 0.10 | | 0.23 | |

Note: LSM: least squares means estimation; OR: odds ratio (between iDCpp65 and control per gender; between iDCpp65 and control irrespective of gender); RR: rate ratio (between iDCpp65 and control per gender; between iDCpp65 and control irrespective of gender).

^1^P-value less than 0.05 is indicated by black and italic.
