# Supplementary material for: Multidimensional Analysis Integrating Human T-Cell Signatures in Lymphatic Tissues with Sex of Humanized Mice for Prediction of Responses after Dendritic Cell Immunization
Source: Front Immunol. 2017 Dec 8;8:1709. doi: 10.3389/fimmu.2017.01709 (PMC5727047; doi:10.3389/fimmu.2017.01709)
Supplement: Supplementary file 5 [file Table_5.docx]

**Supplementary Table 5. Least squares means estimation of mean relative frequency for the analysis of “Combined peripheral and mesenteric LNs” and “Mesenteric LNs” data.**

|  | **Combined peripheral and mesenteric LNs** | | | | | | | **Mesenteric LNs** | | | | | | | |
| --- | --- | --- | --- | --- | --- | --- | --- | --- | --- | --- | --- | --- | --- | --- | --- |
|  | **Female. n=9** | | **Male, n=11** | | | **Group** | | **Female, n=9** | | | **Male, n=12** | | **Group** | | |
|  | **iDCpp65**  **n=6** | **Control**  **n=3** | **iDCpp65**  **n=6** | **Control**  **n=4** | | **iDCpp65 n=12** | **Control n=7** | **iDCpp65 n=5** | **Control n=4** | | **iDCpp65 n=6** | **Control n=6** | **iDCpp65 n=11** | | **Control n=10** |
| **CD19** |  | | | | | | |  | | | | | | | |
| **LSM** | 30.10 | 17.05 | 30.11 | 23.12 | | 30.15 | 20.40 | 38.91 | 20.85 | | 32.48 | 30.78 | 35.46 | | 26.68 |
| **OR** | 2.09 | | 1.43 | | | 1.68 | | 2.42 | | | 1.08 | | 1.51 | | |
| **p-value^1^** | ***0.04*** | | 0.21 | | | ***0.02*** | | ***0.01*** | | | 0.76 | | 0.06 | | |
|  |  | | | | | | |  | | | | | | | |
| **Other CD45^+^** |  | |  | | |  | |  | | |  | |  | | |
| **LSM** | 7.47 | 13.37 | 4.85 | | 8.14 | 6.22 | 10.28 | 9.61 | 14.43 | | 6.65 | 9.19 | 8.07 | | 0.11 |
| **OR** | 0.52 | | 0.57 | | | 0.58 | | 0.63 | | | 0.70 | | 0.69 | | |
| **p-value** | ***0.01*** | | ***0.04*** | | | ***0.01*** | | 0.07 | | | 0.17 | | 0.08 | | |
|  |  | | | | | | |  | | | | | | | |
| **CD3** |  | |  | | |  | |  | | |  | |  | | |
| **LSM** | 62.56 | 69.58 | 64.46 | 68.57 | | 63.51 | 69.00 | 51.92 | | 64.99 | 60.50 | 59.96 | 56.61 | | 61.94 |
| **OR** | 0.73 | | 0.83 | | | 0.78 | | 0.58 | | | 1.02 | | 0.80 | | |
| **p-value** | 0.27 | | 0.47 | | | 0.20 | | 0.06 | | | 0.92 | | 0.23 | | |
|  |  | | | | | | |  | | | | | | | |
| **CD4** |  | |  | | |  | |  | | |  | |  | | |
| **LSM** | 44.39 | 55.36 | 48.93 | 56.24 | | 46.66 | 55.86 | 35.69 | | 43.99 | 43.76 | 45.05 | 40.05 | | 44.65 |
| **OR** | 0.64 | | 0.75 | | | 0.69 | | 0.71 | | | 0.95 | | 0.83 | | |
| **p-value** | 0.20 | | 0.34 | | | 0.12 | | 0.21 | | | 0.82 | | 0.31 | | |
|  |  | | | | | | |  | | | | | | | |
| **CD4N** |  | |  | | |  | |  | | |  | |  | | |
| **LSM** | 15.83 | 19.40 | 10.80 | 17.14 | | 13.21 | 18.24 | 25.31 | | 23.88 | 22.35 | 22.61 | 23.68 | | 23.14 |
| **OR** | 0.78 | | 0.59 | | | 0.68 | | 1.08 | | | 0.98 | | 1.03 | | |
| **p-value** | 0.63 | | 0.29 | | | 0.30 | | 0.88 | | | 0.97 | | 0.93 | | |
|  |  | | | | | | |  | | | | | | | |
| **CD4CM** |  | |  | | |  | |  | | |  | |  | | |
| **LSM** | 30.69 | 37.41 | 47.82 | 40.60 | | 39.12 | 39.44 | 26.31 | | 32.76 | 35.13 | 28.53 | 31.04 | | 30.30 |
| **OR** | 0.74 | | 1.34 | | | 0.99 | | 0.73 | | | 1.36 | | 1.03 | | |
| **p-value** | 0.22 | | 0.17 | | | 0.95 | | 0.39 | | | 0.32 | | 0.89 | | |
|  |  | | | | | | |  | | | | | | | |
| **CD4EM** |  | |  | | |  | |  | | |  | |  | | |
| **LSM** | 50.54 | 36.16 | 40.68 | 34.37 | | 45.59 | 35.23 | 42.54 | 39.05 | | 36.55 | 41.28 | 39.25 | | 40.40 |
| **OR** | 1.80 | | 1.31 | | | 1.54 | | 1.16 | | | 0.82 | | 0.95 | | |
| **p-value** | 0.10 | | 0.39 | | | 0.08 | | 0.73 | | | 0.58 | | 0.86 | | |
|  |  | | | | | | |  | | | | | | | |
| **CD4TE** |  | |  | | |  | |  | | |  | |  | | |
| **LSM** | 4.52 | 4.68 | 2.72 | 3.43 | | 3.59 | 0.40 | 7.55 | 5.74 | | 5.17 | 5.56 | 6.19 | | 5.70 |
| **OR** | 0.96 | | 0.79 | | | 0.89 | | 1.34 | | | 0.92 | | 1.09 | | |
| **p-value** | 0.92 | | 0.57 | | | 0.70 | | 0.48 | | | 0.84 | | 0.76 | | |
|  |  | | | | | | |  | | | | | | | |
| **CD8** |  | |  | | |  | |  | | |  | |  | | |
| **LSM** | 15.80 | 16.82 | 13.28 | 11.93 | | 14.59 | 13.94 | 14.90 | 18.05 | | 15.00 | 12.72 | 15.05 | | 14.75 |
| **OR** | 0.93 | | 1.13 | | | 1.05 | | 0.79 | | | 1.21 | | 1.02 | | |
| **p-value** | 0.75 | | 0.61 | | | 0.77 | | 0.31 | | | 0.36 | | 0.89 | | |
|  |  | | | | | | |  | | | | | | | |
| **CD8N** |  | |  | | |  | |  | | |  | |  | | |
| **LSM** | 22.74 | 27.08 | 19.12 | 27.60 | | 20.90 | 27.41 | 34.26 | 39.13 | | 41.77 | 30.62 | 38.36 | | 33.99 |
| **OR** | 0.79 | | 0.62 | | | 0.70 | | 0.81 | | | 1.63 | | 1.21 | | |
| **p-value** | 0.64 | | 0.31 | | | 0.30 | | 0.68 | | | 0.27 | | 0.57 | | |
|  |  | | | | | | |  | | | | | | | |
| **CD8CM** |  | |  | | |  | |  | | |  | |  | | |
| **LSM** | 14.00 | 34.00 | 27.01 | 31.14 | | 20.16 | 32.68 | 14.77 | 25.63 | | 17.26 | 22.29 | 16.13 | 23.62 | |
| **OR** | 0.32 | | 0.82 | | | 0.52 | | 0.50 | | | 0.73 | | 0.62 | | |
| **p-value** | ***0.003*** | | 0.49 | | | ***0.02*** | | 0.13 | | | 0.40 | | 0.11 | | |
|  |  | | | | | | |  | | | | | | | |
| **CD8EM** |  | |  | | |  | |  | | |  | |  | | |
| **LSM** | 34.20 | 30.11 | 41.13 | 26.85 | | 37.64 | 28.30 | 25.04 | 23.89 | | 23.16 | 22.83 | 24.01 | 23.31 | |
| **OR** | 1.21 | | 1.90 | | | 1.53 | | 1.06 | | | 1.02 | | 1.04 | | |
| **p-value** | 0.63 | | 0.08 | | | 0.13 | | 0.88 | | | 0.96 | | 0.89 | | |
|  |  | | | | | | |  | | | | | | | |
| **CD8TE** |  | |  | | |  | |  | | |  | |  | | |
| **LSM** | 29.58 | 10.39 | 14.03 | 10.58 | | 21.32 | 11.48 | 30.00 | 13.50 | | 20.15 | 22.76 | 24.64 | 18.95 | |
| **OR** | 3.62 | | 1.38 | | | 2.09 | | 2.74 | | | 0.86 | | 1.40 | | |
| **p-value** | ***0.02*** | | 0.49 | | | 0.06 | | 0.06 | | | 0.71 | | 0.33 | | |

Note: LSM: least squares means estimation; OR: odds ratio (between iDCpp65 and control per gender; between iDCpp65 and control irrespective of gender).

^1^P-value less than 0.05 is indicated by black and italic
