# Supplementary material for: Multidimensional Analysis Integrating Human T-Cell Signatures in Lymphatic Tissues with Sex of Humanized Mice for Prediction of Responses after Dendritic Cell Immunization
Source: Front Immunol. 2017 Dec 8;8:1709. doi: 10.3389/fimmu.2017.01709 (PMC5727047; doi:10.3389/fimmu.2017.01709)
Supplement: Supplementary file 6 [file Table_6.docx]

**Supplementary Table 6. Least squares means estimation of mean relative frequencies and counts for the analysis of Thymus data.**

Note: LSM: least squares means estimation; OR: odds ratio (between iDCpp65 and control per gender; between iDCpp65 and control irrespective of gender).

^1^P-value less than 0.05 is indicated by black and italic

|  | **Thymus, %** | | | | | | **Thymus, #** | | | | | |
| --- | --- | --- | --- | --- | --- | --- | --- | --- | --- | --- | --- | --- |
|  | **Female, n=14** | | **Male, n=13** | | **Group, n=27** | | **Female, n=14** | | **Male, n=13** | | **Group, n=27** | |
|  | **iDCpp65 n=9** | **Control n=5** | **iDCpp65 n=7** | **Control n=6** | **Control n=16** | **iDCpp65 n=11** | **iDCpp65 n=9** | **Control n=5** | **iDCpp65 n=7** | **Control n=6** | **iDCpp65 n=16** | **Control n=11** |
| **DP** |  | |  | |  | |  | |  | |  | |
| **LSM** | 29.86 | 30.44 | 48.54 | 20.66 | 37.74 | 25.39 | 452392 | 528744 | 468816 | 203668 | 459577 | 351430 |
| **OR/RR** | 0.97 | | 3.62 | | 1.78 | | 0.85 | | 2.30 | | 1.31 | |
| **p-value^1^** | 0.96 | | 0.054 | | 0.21 | | 0.82 | | 0.29 | | 0.61 | |
|  |  | |  | |  | |  | |  | |  | |
| **CD4SP** |  | |  | |  | |  | |  | |  | |
| **LSM** | 27.18 | 29.70 | 21.14 | 33.62 | 24.48 | 31.88 | 147961 | 335960 | 163154 | 144562 | 154608 | 231561 |
| **OR/RR** | 0.88 | | 0.53 | | 0.69 | | 0.44 | | 1.13 | | 0.67 | |
| **p-value** | 0.75 | | 0.12 | | 0.19 | | 0.03 | | 0.71 | | 0.19 | |
|  |  | |  | |  | |  | |  | |  | |
| **CD8SP** |  | |  | |  | |  | |  | |  | |
| **LSM** | 16.37 | 19.56 | 16.57 | 17.49 | 16.47 | 18.41 | 86111 | 205875 | 146197 | 73379 | 112399 | 133604 |
| **OR/RR** | 0.80 | | 0.94 | | 0.87 | | 0.42 | | 1.99 | | 0.84 | |
| **p-value** | 0.50 | | 0.84 | | 0.56 | | ***0.002*** | | 0.10 | | 0.59 | |
|  |  | |  | |  | |  | |  | |  | |
| **DN** |  | |  | |  | |  | |  | |  | |
| **LSM** | 20.21 | 15.39 | 10.72 | 21.54 | 15.72 | 19.04 | 90537 | 115521 | 60451 | 90113 | 77374 | 101662 |
| **OR/RR** | 1.39 | | 0.44 | | 0.79 | | 0.78 | | 0.67 | | 0.76 | |
| **p-value** | 0.49 | | 0.10 | | 0.51 | | 0.34 | | 0.41 | | 0.28 | |
|  |  | |  | |  | |  | |  | |  | |
