# Supplementary material for: Multidimensional Analysis Integrating Human T-Cell Signatures in Lymphatic Tissues with Sex of Humanized Mice for Prediction of Responses after Dendritic Cell Immunization
Source: Front Immunol. 2017 Dec 8;8:1709. doi: 10.3389/fimmu.2017.01709 (PMC5727047; doi:10.3389/fimmu.2017.01709)
Supplement: Supplementary file 7 [file Table_7.docx]

**Supplementary Table 7. Results of ANN classification**

| **%** | **Tissues (based on cell frequencies)** | | | | | | | | | | | | | | | | | |
| --- | --- | --- | --- | --- | --- | --- | --- | --- | --- | --- | --- | --- | --- | --- | --- | --- | --- | --- |
|  | **Mesenteric + Peripheral**  **LNs (n=19)** | | | **Mesenteric LNs**  **(n=18)** | | | **Spleen**  **(n=25)** | | | **Peripheral Blood**  **(n=28 )** | | | **Bone Marrow**  **(n=28 )** | | | **Thymus**  **(n= 27)** | | |
| **Classification, %**  **(Std. dev.)** | ***Accuracy*** | *Sensitivity* | *Specificity* | ***Accuracy*** | *Sensitivity* | *Specificity* | ***Accuracy*** | *Sensitivity* | *Specificity* | ***Accuracy*** | *Sensitivity* | *Specificity* | ***Accuracy*** | *Sensitivity* | *Specificity* | ***Accuracy*** | *Sensitivity* | *Specificity* |
| **Combined** | 71.1  (17.1) | 83.1  (29.7) | 50.6  (39.97) | 67.57  (16.1) | 78.2  (31.2) | 50.2  (40.96) | 70.6  (15.2) | 85.8  (27.8) | 38.2  (38.8) | 73.3  (17.8) | 84.7  (28.5) | 55.7  (41.9) | 65.2  (13.6) | 75.6  (31.3) | 42.95  (37.9) | 63.75  (13.2) | 81.0  (31.2) | 38.7  ( 31.2) |
| **Females** | 77.3  (22.5) | 84.5  (31.1) | 62.9  (43.1) | 74.4  (22.96) | 79.5  (34.6) | 65.9  (40.7) | 72.4  (18.3) | 84.5  (29.9) | 45.0  (39.97) | 68.9  (17.2) | 80.0  (30.6) | 48.9  (40.9) | 71.97  (18.6) | 83.3  (30.4) | 51.6  (40.4) | 61.4  (13.4) | 72.9  (33.4) | 40.7  (38.9) |
| **Males** | 66.7  (18.8) | 74.2  (34.5) | 55.6  (39.5) | 60.2  (14.8) | 67.3  (34.9) | 49.6  (39.2) | 70.1  (18.7) | 80.4  (31.5) | 49.5 (41.1) | 70.6  (19.4) | 78.1  (33.0) | 60.6  (40.9) | 60.2  (13.8) | 67.3  (36.6) | 50.6  (39.8) | 62.5  (15.4) | 71.3  (36.6) | 52.16  (37.8) |
