# Supplementary material for: Multidimensional Analysis Integrating Human T-Cell Signatures in Lymphatic Tissues with Sex of Humanized Mice for Prediction of Responses after Dendritic Cell Immunization
Source: Front Immunol. 2017 Dec 8;8:1709. doi: 10.3389/fimmu.2017.01709 (PMC5727047; doi:10.3389/fimmu.2017.01709)
Supplement: Supplementary file 8 [file Presentation_1.pptx]

## Slide 1
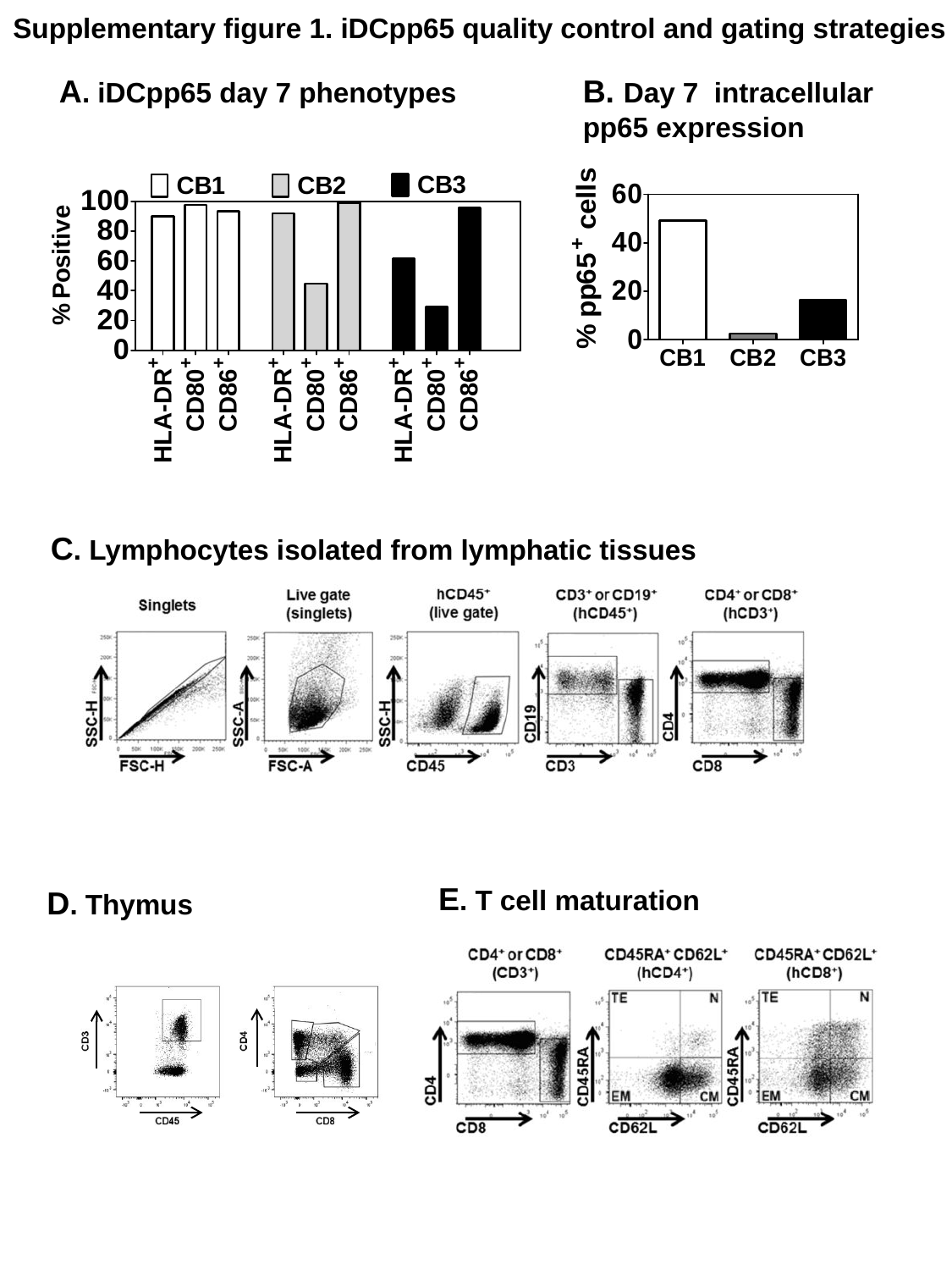

Supplementary figure 1. iDCpp65 quality control and gating strategies
A. iDCpp65 day 7 phenotypes
B. Day 7 intracellular
pp65 expression
 C. Lymphocytes isolated from lymphatic tissues
 E. T cell maturation
 D. Thymus
