# Supplementary material for: Multidimensional Analysis Integrating Human T-Cell Signatures in Lymphatic Tissues with Sex of Humanized Mice for Prediction of Responses after Dendritic Cell Immunization
Source: Front Immunol. 2017 Dec 8;8:1709. doi: 10.3389/fimmu.2017.01709 (PMC5727047; doi:10.3389/fimmu.2017.01709)
Supplement: Supplementary file 9 [file Presentation_2.pptx]

## Slide 1
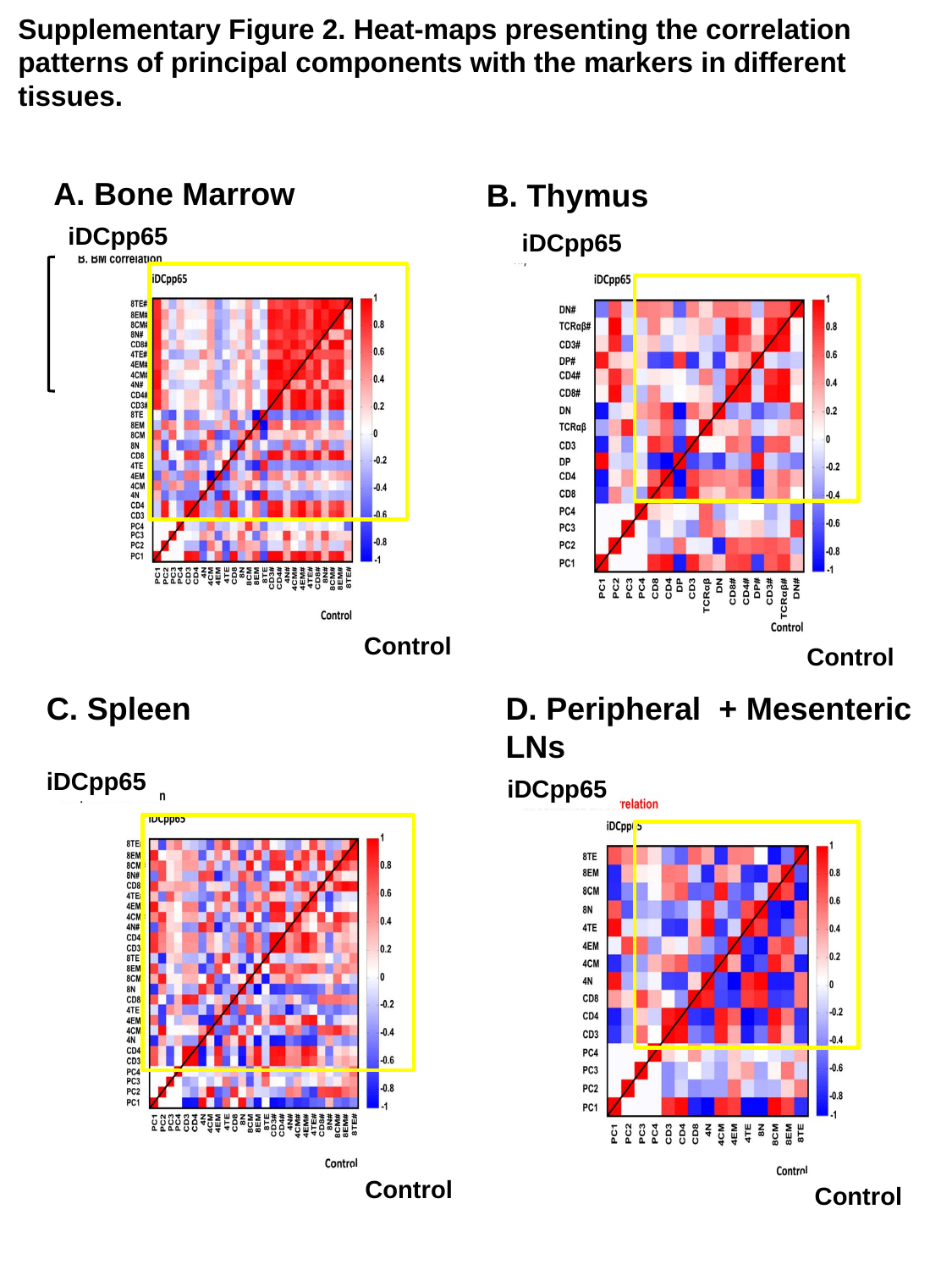

Supplementary Figure 2. Heat-maps presenting the correlation patterns of principal components with the markers in different tissues.
A. Bone Marrow
B. Thymus
iDCpp65
iDCpp65
Control
Control
C. Spleen
iDCpp65
Control
D. Peripheral + Mesenteric
LNs
iDCpp65
Control
